# The combination of neoantigen quality and T lymphocyte infiltrates identifies glioblastomas with the longest survival

**DOI:** 10.1038/s42003-019-0369-7

**Published:** 2019-04-23

**Authors:** Jing Zhang, Francesca P. Caruso, Jason K. Sa, Sune Justesen, Do-Hyun Nam, Peter Sims, Michele Ceccarelli, Anna Lasorella, Antonio Iavarone

**Affiliations:** 1 Institute for Cancer Genetics, Columbia University Medical Center, New York, NY 10032 USA; 2 Department of Science and Technology, Universita’ degli Studi del Sannio, 82100 Benevento, Italy; 3 BIOGEM Istituto di Ricerche Genetiche ‘G. Salvatore’, Campo Reale, 83031 Ariano Irpino, Italy; 4 Institute for Refractory Cancer Research, Samsung Medical Center, Seoul, Republic of Korea; 5 Immunitrack Aps, Rønnegade 4, 2100 Copenhagen East, Denmark; 6 Department of Health Sciences and Technology, SAIHST, Sungkyunkwan University, Seoul, Republic of Korea; 7 Department of Neurosurgery, Samsung Medical Center, Sungkyunkwan University School of Medicine, Seoul, Republic of Korea; 8 Department of Systems Biology, Columbia University Medical Center, New York, NY 10032 USA; 9 ABBVIE, Redwood City (CA), Redwood City, CA 94063 USA; 10 Department of Pediatrics, Columbia University Medical Center, New York, NY 10032 USA; 11 Department of Pathology and Cell Biology, Columbia University Medical Center, New York, NY 10032 USA; 12 Department of Neurology, Columbia University Medical Center, New York, NY 10032 USA

**Keywords:** Tumour immunology, CNS cancer

## Abstract

Glioblastoma (GBM) is resistant to multimodality therapeutic approaches. A high burden of tumor-specific mutant peptides (neoantigens) correlates with better survival and response to immunotherapies in selected solid tumors but how neoantigens impact clinical outcome in GBM remains unclear. Here, we exploit the similarity between tumor neoantigens and infectious disease-derived immune epitopes and apply a neoantigen fitness model for identifying high-quality neoantigens in a human pan-glioma dataset. We find that the neoantigen quality fitness model stratifies GBM patients with more favorable clinical outcome and, together with CD8^+^ T lymphocytes tumor infiltration, identifies a GBM subgroup with the longest survival, which displays distinct genomic and transcriptomic features. Conversely, neither tumor neoantigen burden from a quantitative model nor the isolated enrichment of CD8^+^ T lymphocytes were able to predict survival of GBM patients. This approach may guide optimal stratification of GBM patients for maximum response to immunotherapy.

## Abstract (web summary)

Jing Zhang et al. analyze glioblastoma patient data with a computational model integrating mutation-derived neoantigens similar to pathogen-specific antigens with enrichment of signatures for individual immune cells. Using a fitness model for identifying high-quality neoantigens and T lymphocyte infiltrates in glioblastoma, they uncover a subset of patients with prolonged survival.

## Introduction

Recent reports have shown that nonsynonymous coding mutations may increase tumor immunogenicity. In selected tumor types such as melanoma, lung cancer, and colorectal tumors, the somatic mutational burden correlates with the probability to generate immunogenic peptides that are presented to CD8^+^ T cells on restricted HLA-I subtypes^1–4^. However, in most solid tumors, T-cell immunity and a productive response to immune therapies have been reported only in a minority of patients and the identity of immunogenic tumor antigens, also referred to as neoantigens, remains unknown.

High-grade glioma is the most frequent type of primary brain tumor, with grade IV glioma (glioblastoma, GBM) being an invariably lethal tumor type with median survival below 15 months^5,6^. In the case of glioma higher mutational load is associated with increased tumor aggressiveness^7^. Consequently, the role, if any, of mutation-generated neoantigens as inducers of immunogenic responses in GBM has remained elusive. A further element that limits a productive anti-tumor immunity in GBM is the tumor microenvironment, which is dominated by myeloid-derived cells, mostly blood-derived macrophages and resident microglia^8–11^ actively operating to exclude T lymphocytes and undermine their function^12^. Accordingly, GBM typically lack significant number of T lymphocyte infiltrates^13^. The recognized unique genetic landscape and the biological features of the GBM microenvironment led to the exclusion of high-grade glioma patients from several multi-cancer studies that have characterized tumor immunity and reinforced the notion that a lymphocyte depleted and immunosuppressive microenvironment is a distinctive feature of malignant gliomas^14^.

In this manuscript, we present the application of a neoantigen quality model for the accurate prediction of immunogenic neoantigens in IDH wild-type GBM, the largest and most aggressive group of high-grade gliomas. We found that in IDH wild-type GBM the production of high-quality neoantigens and infiltration of T lymphocytes are distinctive features of patients with the longest survival. The unique immunogenic attributes of this GBM subgroup informs on a cohort of patients who are optimally outfitted to mount the most effective responses following immunotherapy treatments.

## Results

### Neoantigen quantity fails to predict survival of gliomas

**Fig. 1:**
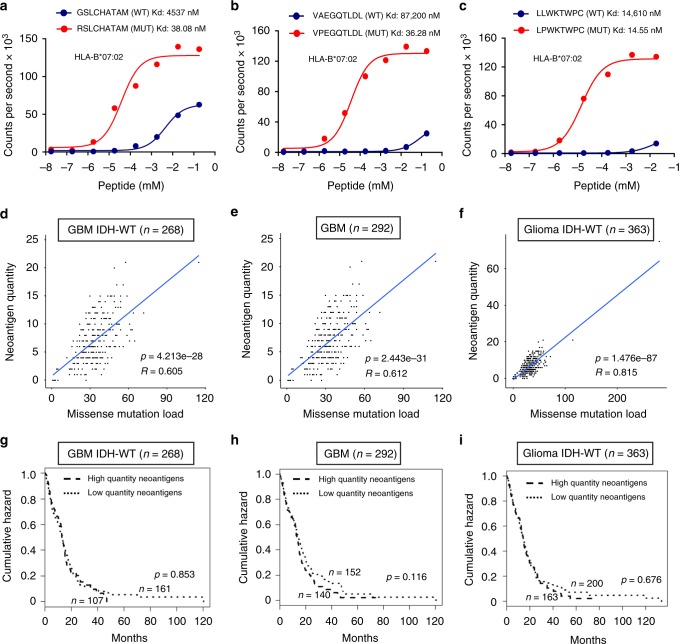
Neoantigen quantity is not prognostic of survival in glioma. **a**–**c** In vitro binding affinity kinetics of neoantigens and corresponding wild-type peptides for their restricted HLA class I allele. Representative results for **a**, GBM IDH wild-type; **b**, GBM; **c**, glioma IDH wild-type. Data are shown as counts per second with increasing peptide concentration (log_10_ mM). Data are mean of *n* = 4 technical replicates from two independent experiments for wild-type peptide; *n* = 2 technical replicates for mutant peptide (**a**, **c**); *n* = 4 technical replicates from two independent experiments for wild-type peptide; *n* = 6 technical replicates from two independent experiments for mutant peptide (**b**). **d**–**f** Analysis of the correlation between neoantigen quantity and missense mutation load; **d**, GBM IDH wild-type; **e**, GBM; **f**, glioma IDH wild-type. **g**–**i** Stratification of survival according to neoantigen quantity score; dashed line: high-quantity neoantigens; dotted line: low-quantity neoantigens. **g**, GBM IDH wild-type; **h**, GBM; **i**, glioma IDH wild-type. *n* is the number of patients. *p*-value was determined using a log-rank test

To define the importance of neoantigens in human glioma, we designed a stringent neoantigen prediction algorithm that considers the differential binding affinity of mutant and wild-type 9-mer peptides to HLA-I (neoantigen quantity model, Supplementary Fig. 1). Binding affinity was determined by netMHCpan-4.0^15^. Only mutant peptides with binding affinity IC50 < 500 nM for the restricted HLA-I subtype and binding affinity of the corresponding wild-type peptide IC50 > 500 nM for all HLA-I subtypes were retained as neoantigens (Supplementary Figs. 1–3). HLA-I subtyping for each glioma patient was done through the implementation of four different algorithms (PolySolver^16^, OptiType^17^, PHLAT^18^, and seq2HLA^19^). We applied the neoantigen prediction algorithm to the ATLAS-TCGA pan-glioma cohort that includes 303 GBM and 509 lower-grade glioma (LGG) profiled by whole exome sequence (WES), RNA-seq, and Agilent transcriptomic array. Clinical annotations were also available for this cohort (Supplementary Data 1). The pan-glioma cohort had been previously stratified on the basis of histology, genetic alterations, DNA methylation, and transcriptome clustering resulting in 19 glioma subgroups with distinct overlaps (Supplementary Data 1)^20^. First, we calculated the HLA-I allele frequency in each glioma subtype and found that different glioma subtypes have similar HLA-I alleles frequency (Benjamini–Hochberg corrected Fisher exact test *p* value > 0.05). We characterized neoantigens and immune landscape for each glioma subgroup, stratified tumors into high and low-neoantigen groups on the basis of the mean value of the neoantigen load and compared survival by Kaplan–Meier analysis. As experimental validation of the neoantigen prediction, we used a homogenous, proximity-based luminescent oxygen channeling immunoassay to determine the affinity kinetics of the predicted glioma neoantigens for binding to HLA-I subtypes^21^. This analysis including 14 matched glioma neoantigens and corresponding wild-type peptides revealed that each mutant peptide bound with higher affinity to HLA-I than the wild-type counterpart, thus validating the stringency of our approach (Fig. 1a–c, Supplementary Fig. 4). However, neoantigen load, which correlated with mutational load across glioma subtypes (Fig. 1d–f), did not distinguish patients according to clinical outcome in the cohort of GBM IDH wild-type, GBM, glioma IDH wild-type or the most aggressive form of glioma (mesenchymal and classical), but a higher neoantigen load was associated with worse prognosis in lower grade gliomas (with or without co-deletion of chromosome 1p and chromosome 19q and regardless of histology) and in glioma of the proneural and neural subtype (Fig. 1g–i, Supplementary Fig. 5). Recently, it has been proposed that the difference in binding affinity between any wild-type and mutant peptide (termed differential agretopicity index, DAI^22,23^) is a more accurate indicator of peptide immunogenicity than the binding affinity of the mutant peptide and it has been shown that the mean DAI of all tumor peptide pairs was a predictor of survival in melanoma and non-small cell lung cancer^24^. We calculated mean DAI for each glioma in the TCGA cohort and determined that similar to the results obtained from the quantity model, patients in different glioma sub-groups with high (above mean) or low (below mean) DAI had similar prognosis (Supplementary Fig. 6). In some glioma sub-types and the aggregated cohort of all gliomas, high DAI was associated with a worse clinical outcome (Supplementary Fig. 6). Together, these findings suggest that in contrast to other cancer types^25,26^, the neoantigen load is only a representation of the tumor mutation burden and is not associated with better survival.

### High-quality neoantigens predict better survival in GBM

Similarity to known pathogen immunogens has been used to define neoantigen fitness (neoantigen quality model) and appears to provide a more accurate prediction of anti-tumor immunity and patients survival than the neoantigen load^27^. We postulated that T cell receptors (TCRs) that can recognize pathogenic antigens can also recognize similar non-pathogenic neoantigens generated by glioma cells and applied a neoantigen fitness model to glioma. In this model the candidate neoantigens are used to compute the Neoantigen Recognition Potential (NRP) which is the product of two terms (Supplementary Fig. 7 and Methods). The first term approximates the probability that a presented neoantigen will be recognized by the TCR repertoire and it depends on the similarity of the mutant peptide to human infectious disease-derived peptide sequences with positive immune assays in the Immune Epitope Database (IEDB)^28^. The second term, defined as the amplitude and similar to the DAI, accounts for the ratio between the binding probabilities of wild-type and mutant peptides. This model was successfully applied to predict response to checkpoint blockade immune-therapy in melanoma and lung cancer^29^. The first term of NRP depends on the parameters of a logistic model that were optimized for best stability and robustness across the major subtypes of high-grade gliomas (Supplementary Fig. 8). We discovered that NRP is significantly higher in IDH wild-type gliomas when compared with IDH mutant gliomas (Mann–Whitney *U* test *p*-value = 2.82e–13, Supplementary Fig. 9a).

**Fig. 2:**
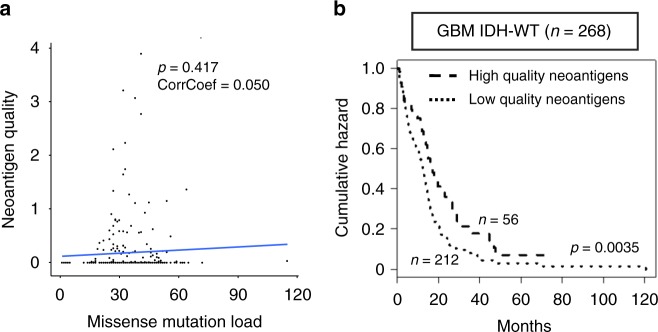
High-quality neoantigens are prognostic of better survival in IDH wild-type GBM. **a** Lack of correlation between neoantigen quality and missense mutation load (*R* = 0.050, *p* = 0.417). **b** Stratification of survival according to neoantigen quality score (log rank test *p* = 0.0035)

The application of the quality model analysis, which is independent of mutation burden (Fig. 2a), to 268 IDH wild-type GBM patients, revealed that high-quality neoantigen score (above the mean values) was associated with a significantly longer survival (Fig. 2b). This effect was independent of age, gender, and mutation load (Supplementary Table 1). Similarly, stratification by the mean value of total neoantigen quality separated the cohorts of GBM (including 292 patients) and IDH wild-type gliomas (including 363 patients) into two distinct prognostic sub-groups (Supplementary Fig. 10a, b). A non-statistically significant trend to better survival for high-quality neoantigens was also observed in classical, classic-like, and mesenchymal glioma (Supplementary Fig. 10c–e). We also determined that neoantigens with high-quality score were not restricted to specific HLA-I subtypes in IDH wild-type GBM patients (Benjamini–Hochberg corrected proportion test, *p* value > 0.05). As an independent validation of the quality model, we used WES from 46 primary GBMs from a recently published cohort for which we obtained most updated survival data^30^. We confirmed that the 15 patients with tumors predicted to contain high-quality neoantigens had a significantly better survival (log rank *p* = 0.0339, Supplementary Fig. 11).

As IDH wild-type tumors compose the largest and most homogeneous subgroup of GBM and exhibit optimal performance in the glioma fitness model analysis, we focused our subsequent analyses on this group of tumors. HLA class I molecules are highly polymorphic with variation located in the peptide-binding region, with each variant binding only to a highly restricted set of peptide ligands^31^. Compared to heterozygous HLA-I carriers, homozygosity for HLA-I loci is predicted to present a smaller and less diverse repertoire of tumor-derived neoantigens to cytotoxic T lymphocytes (CTLs)^32^. We therefore asked whether greater diversity (heterozygosity) in the repertoire of antigen-presenting HLA-I molecules is associated with more efficient recognition of high-quality neoantigens. The analysis of the variations at each of the HLA-I genes (HLA-A, HLA-B, and HLA-C) in the high and low-quality neoantigen cohorts revealed that at least one HLA-I allele was homozygous in 25.0% (53 of 212) of IDH wild-type GBM containing only low-quality neoantigens but the frequency of HLA-I homozygosity was significantly lower in the high-quality neoantigen group (10.7% or 6 of 56, One-sided Fisher exact test *p* = 0.0136, Supplementary Table 2). Although not reaching statistical significance, also the amplitude term of neoantigens in HLA-I homozygosity was inferior to the amplitude term of neoantigens in HLA-I heterozygosity (median amplitude term: 1.583 and 1.785 for HLA-I homozygosity and HLA-I heterozygosity, respectively; Mann–Whitney *U* test *p* value = 0.176, Supplementary Fig. 12a). We also determined that the number of neoantigens in HLA-I homozygous IDH wild-type GBM is significantly lower than HLA-I heterozygous patients (Mann–Whitney *U* test *p* value = 0.0001, Supplementary Fig. 12b). Together, these results indicate that homozygosity for HLA-I alleles hinders the ability to generate and recognize high-quality neoantigens.

**Fig. 3:**
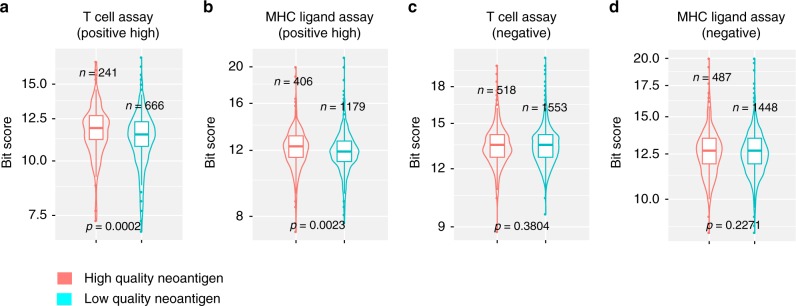
High-quality neoantigens are associated with immunogenicity in IDH wild-type GBM. Comparison of the similarity between neoantigens and human immune epitopes scored as immunogenic (positive high) or non-immunogenic (negative) in high and low-quality neoantigen groups of IDH wild-type GBM. **a**, **c** T cell assays; **b**, **d** MHC ligand assays. *n* number of neoantigens. *p*-value was determined using two-sided Mann–Whitney *U* test

As the IEDB database also contains a collection of human immune epitopes tested with experimental assays and annotated for their ability to trigger an immune response, we sought to provide an independent validation of the quality of the computationally identified neoantigens in human gliomas. We aligned the sequences of the neoantigens detected in the high and low-quality neoantigen group of IDH wild-type GBM with experimentally validated human epitopes (allergy and autoimmune-derived) in IEDB. The analysis showed that, in comparison with low-quality neoantigens, high-quality neoantigens exhibited greater similarity to human peptides in IEDB that score as highly immunogenic (“positive high”) in T cell and MHC ligand assays (*p* = 0.0002 and *p* = 0.0023, respectively; Fig. 3a, b). Conversely, we found no difference between low and high-quality neoantigens in the alignment with human peptides in IEDB that score negative in validation immune assays (*p* = 0.3804 and 0.2271, respectively, Fig. 3c, d). Taken together, neoantigen quality rather than neoantigen load correlates with immunogenicity and predicts survival in IDH wild-type GBM.

### High-quality neoantigens and CD8^+^ T cells identify the longest survivors

**Fig. 4:**
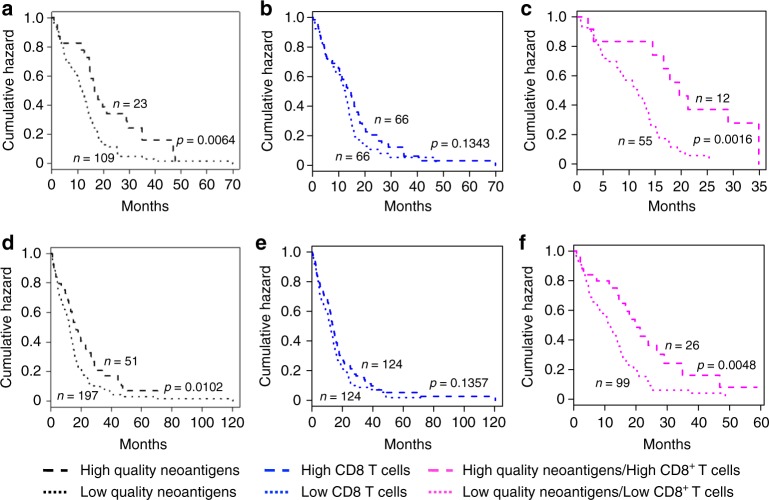
Synergistic effect of CD8^+^ T cells and high-quality neoantigens on survival of IDH wild-type GBM. **a**–**c** Analysis of the cohort for which WES and RNAseq were available. **a** Survival of patients stratified according to neoantigen quality score. **b** Survival of patients stratified according to CD8^+^ T lymphocyte enrichment score. **c** Survival of patients stratified by neoantigen quality and CD8^+^ T lymphocytes infiltration score. **d**–**f** Analysis of the cohort for which WES and Agilent microarray were available. **d** Survival of patients stratified according to neoantigen quality score. **e** Survival of patient stratified according to CD8^+^ T lymphocyte enrichment score. **f** Survival of patients stratified by neoantigen quality and CD8^+^ T lymphocytes infiltration score. Black dashed and dotted lines represent samples with high and low-quality neoantigens, respectively. Blue dashed and dotted lines represent patients with high and low CD8^+^ T lymphocytes, respectively. Pink dashed and dotted lines represent patients with high-quality neoantigens and high CD8^+^ T lymphocytes and low-quality neoantigens and low CD8^+^ T cells, respectively. *n* number of patients. *p-*value was determined by the log-rank test

Having established that high-quality neoantigens improve survival of IDH wild-type GBM patients, we turned our attention to the role of the non-tumor cells that compose the GBM microenvironment. In particular, we sought to determine whether the presence of any of the non-tumor cell populations (immune and non-immune) that infiltrate human GBM impact survival and/or cooperate with high-quality neoantigens to determine a more favorable outcome of GBM patients. The GBM tumor microenvironment is dominated by myeloid-derived cells that can be separated into blood-derived macrophages and microglia. We recently reported the single cell transcriptome of 8 GBM^33^. The analysis of cells of the tumor microenvironment from this study using single cell MWW-Gene Set Test (MWW-GST)^34,35^ led to the identification of 6 distinct cell populations (blood-derived macrophages, microglia, CD8^+^ T lymphocytes, oligodendrocytes, endothelial cells and pericytes). For each cell type, we selected a specific signature of 30 genes and calculated the median enrichment score in order to separate IDH wild-type GBM patients into groups with high or low infiltration of individual cell populations and asked whether the enrichment of specific cell types was associated with changes in survival. We also computed the individual signature of 12 previously identified tumor infiltrating immune cells, thus including a total of 18 cell type-specific signatures plus two signatures for the interferon gamma response pathway (Supplementary Data 2)^33,36–39^_._ The analysis was performed on 132 and 248 TCGA-derived GBM that had been analyzed by RNA-seq or Agilent expression arrays, respectively. Whereas we detected a significant effect on survival of some cell populations in one of the cohorts, none of the 20 signatures concordantly differentiated the survival of GBM patients in both cohorts (Supplementary Table 3). Infiltration of CD8^+^ T lymphocytes that has been shown to predict a favorable prognosis in several tumor types^40–43^ was associated only with a non-statistically significant trend to a better clinical outcome in the RNAseq and Agilent microarray cohorts (*p* = 0.134 and *p* = 0.136, respectively; Fig. 4b, e). Confirming the lack of significance of CD8^+^ T cell enrichment score for survival, we found a lower CD8^+^ T cell enrichment score in the more favorable IDH mutant when compared with IDH wild-type gliomas (Wilcoxon *p*-value 2.26E–16, Supplementary Fig. 9b). When IDH wild-type GBM patients were stratified according to the enrichment of each non-tumor cell type in combination with high or low-quality neoantigens, elevated CD8^+^ T lymphocytes and high-quality neoantigens emerged as the only features that induced a synergistic effect on survival and classified a subgroup of approximately 10% of IDH wild-type GBM patients with the longest survival in both RNAseq and Agilent microarray datasets (*p* = 0.0016 and *p* = 0.0048, respectively; Fig. 4a–f and Supplementary Table 3). The positive role of CD8^+^ T cells in the synergy with high-quality neoantigens was recapitulated by the trend for a similar cooperation towards a better clinical outcome between high-quality neoantigens and the interferon gamma signature, which is used as a surrogate of CD8^+^ T cell function in transcriptomic analyses^38,39,44^ (Supplementary Table 3). The synergistic effect of high-quality neoantigens and CD8^+^ T lymphocyte activation was still significant in a multivariate regression model including age, gender and mutational load as additional covariates (Supplementary Table 4). To determine the relationship between T cell infiltration, high-quality neoantigens and tumor purity, we used the tumor purity values calculated by ABSOLUTE^45^, a validated computational approach for the inference of the fraction of stromal/immune cells and consequently tumor cell purity. As expected, we found a negative correlation between CD8^+^ T cell enrichment score and tumor purity (correlation coefficient = −0.457, *p* = 3.695e–8, Supplementary Fig. 13a). However, there was no correlation between neoantigen quality score and tumor purity (correlation coefficient = 0.0839, *p* = 0.186, Supplementary Fig. 13b), indicating that high-quality neoantigens are independent of broad immune cell infiltration. Aberrant DNA methylation of genes expressed by immune cells were reported to regulate the extent of immune infiltration in solid tumors^46–48^. Therefore, we examined whether the enrichment of CD8^+^ T cells in IDH wild-type GBM is associated with differential DNA methylation. Towards this aim, we performed an integrated analysis of gene expression and DNA methylation. From this analysis, diverse immune response categories emerged as significantly hypo-methylated and upregulated in IDH wild-type GBM patients with high CD8^+^ T cells for both RNAseq (Supplementary Fig. 14a, b; Supplementary Data 3, 5) and Agilent expression data (Supplementary Fig. 14c, d; Supplementary Data 4, 6). Taken together, the combination of productive neoantigen T cell recognition and epigenetically directed intra-tumor infiltration of CD8^+^ T lymphocytes characterizes a sizable group of IDH wild-type GBM patients who experience a more favorable prognosis.

### High-quality neoantigens and CD8^+^ T cells activate the immune response

**Fig. 5:**
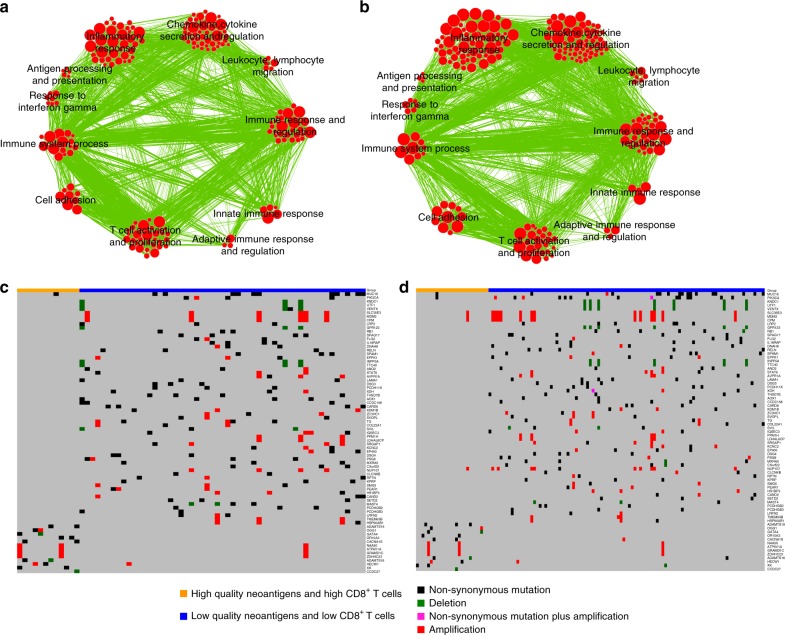
Gene ontology enrichment networks and genetic characteristics of IDH wild-type GBM with high-quality neoantigens and high CD8^+^ T cells. **a** Enrichment map network of statistically significant GO categories in the patient cohort analyzed by WES and RNAseq. **b** Enrichment map network of statistically significant GO categories in the patient cohort with WES and Agilent data available (normalized enrichment score, NES > 0.6, and *q*-value < 0.00001). Nodes represent GO terms and lines their connectivity. Node size is proportional to the number of genes in the GO category and line thickness indicates the fraction of genes shared between groups. **c** Landscape of somatic genomic alterations (non-synonymous mutations, copy number alterations) in IDH wild-type GBM (GBM cohort analyzed by WES and RNAseq). **d** Landscape of somatic genomic alterations (non-synonymous mutations, CNVs) in IDH wild-type GBM (GBM cohort analyzed by WES and Agilent microarrays). Rows and columns represent genes and tumor samples, respectively. Genomic alterations are indicated. Genes are sorted according to frequency (% patients) in patients having both high-quality neoantigens and high CD8^+^ T lymphocytes or patients having both low-quality neoantigens and low CD8^+^ T lymphocytes, respectively

To identify the transcriptomic features of IDH wild-type GBM with high-quality neoantigens and high CD8^+^ T cells, we used the combination of the easy ensemble (ee) undersampling technique and Mann–Whitney–Wilcoxon (MWW) test statistics (ee-MWW) we recently developed^34^ and generated a ranked list of genes from RNAseq and Agilent microarray datasets of IDH wild-type GBM discriminating tumors with high-quality neoantigens/high CD8^+^ T cells from those with low-quality neoantigens/low CD8^+^ T cells. The gene ontology enrichment map network (*Q* < 0.00001, normalized enrichment score, NES > 0.6) revealed that the most significant biological processes enriched in IDH wild-type GBM from both RNAseq and Agilent microarray datasets were immune response categories, thus providing additional evidence for the specific immune functions implemented within the tumors that contain high-quality neoantigens and high CD8^+^ T cells (Fig. 5a, b).

### GBM with high-quality neoantigens and CD8^+^ T cells harbor distinct genetic lesions

Next, we sought to identify the genetic features (mutations and copy number variations, CNVs) that distinguish GBM that generate high-quality neoantigens and high CD8^+^ T lymphocyte signatures. We failed to find recurrent genes harboring mutations that produce neoantigens in the high-quality neoantigens/high CD8^+^ T cell group. Similarly, this group lacked specific recurrent mutations. In contrast, we found that tumors without high-quality neoantigens and CD8^+^ T cells harbored a number of recurrently mutated genes (somatic mutations and CNVs; Fig. 5c, d), some of which are important cancer drivers. In particular, we found that genetic alterations of *PIK3CA*, *RB1* and *MDM2* were present in 24–28% of GBM unable to generate high-quality neoantigens and attract CD8^+^ T lymphocytes but only in 0–8% of GBM with high-quality neoantigens and high CD8^+^ T lymphocytes (RNA-seq, *p* = 0.06; Agilent, *p* = 0.02; Supplementary Table 5). Together with the other genes that were exclusively mutated in the low-quality neoantigens/low CD8^+^ T cell group, these findings point to the set of genetic determinants that should support the prospective exclusion of patients with GBM from the high-quality neoantigens/high CD8^+^ T lymphocytes group.

## Discussion

With neoantigens emerging as attractive targets in the development of personalized immunotherapies, strategies for the rapid identification of relevant neoantigens have become a major priority^24,27,29^. This study describes such strategy using a computational approach for the identification of GBM patients harboring high-quality neoantigens that, together with CD8^+^ T lymphocyte infiltrates, perform optimally in identifying patients with the longest survival and a functionally activated tumor immune microenvironment. This information might be of clinical importance for the accurate stratification of the subgroup of GBM patients having the best probability to benefit from immunotherapies. GBM are classified as lymphocyte-depleted tumors^49^ lacking naïve T cells that are instead found sequestered in large numbers in the bone marrow^50^. Nevertheless, recent studies based on single cell profiling have shown that a small proportion of GBM show CD8^+^ T lymphocyte infiltrates^33^.

Progression from low-grade to high-grade glioma evolves through increasing mutational burden^7^. Even among GBM patients, a higher number of mutations is associated with a more aggressive disease and worse survival^7^. Therefore, whereas in several tumor types the tumor mutational burden is associated with activation of an immune response and better survival, GBM displays an opposite behavior^1,3,26,51–53^. Consistent with this notion, we found that the simple estimate of the mutation-derived neoantigen load using a quantity model and the DAI index failed to segregate sub-groups of patients with distinct clinical outcome. Conversely, a high-quality neoantigen model that evaluates the similarity of tumor antigens with highly immunogenic pathogen-derived antigens emerged from our work as the exclusive parameter that was able to identify patients with IDH wild-type GBM who display a more favorable clinical outcome.

Results from the previous studies that have analyzed the role of CD8^+^ T lymphocytes for GBM survival have been conflicting^54,55^. Some of the studies reported that the presence of T lymphocyte infiltrates was associated with a more favorable clinical course of the disease^56–58^, but others reached opposite conclusions^54,59^. In our analysis, which is based on mRNA expression profile from GBM-derived single T lymphocytes, enrichment of the T cell-specific signature was associated with a weak positive effect on survival. However, when combined with the presence of high-quality neoantigens, CD8^+^ T lymphocyte infiltrates provided the best predictive model for the identification of the longest survivors among IDH wild-type GBM.

We suggest that the combination of high-quality neoantigen fitness model and elevated T lymphocyte-specific gene signature together with histopathological verification of tumor infiltration by CD8^+^ T cells should be used in current clinical trials of IDH wild-type GBM for the identification of those patients who have the highest likelihood of clinical response to immune therapy.

## Methods

### Data preparation and preprocessing

The patient cohort is from the ATLAS-TCGA pan-glioma study^20^, which includes 1122 glioma patients with clinical information. For 812 glioma patients with tumor and matched normal samples 30,729 somatic mutations were called using exome-seq data bam files from TCGA Data Portal (http://tcga-data.nci.nih.gov/tcga/)^20^ using at least two of three methods, MuTect^60^, VarScan^61^, and RADIA^62^.

RNA-seq raw counts of 667 cases (513 LGG and 154 GBM) were downloaded, normalized, and filtered using the Bioconductor package TCGAbiolinks^63^ including TCGAquery, TCGAdownload, and TCGAprepare for level 3 data from platform “IlluminaHiSeq_RNASeqV2”. The union of the two matrices (LGG and GBM) was then normalized using within-lane normalization to adjust for GC-content effect on read counts and upper-quantile between-lane normalization for distributional differences between lanes by applying the TCGAanalyze_Normalization function encompassing EDASeq protocol^20^.

Gene expression microarray data with Agilent chip (G4502A) at level 3 were downloaded from TCGA Data Portal. The gene expression data matrix includes 583 samples (573 GBM and 10 normal brains) and 17,814 genes.

Data from 1084 glioma patients with tumor and normal samples that had been profiled on Affymetrix SNP6.0 GeneChip arrays, processed into genome segmentation files^64^ and analyzed by GISTIC2.0 to identify focal copy number changes^20,65^ were downloaded from TCGA Data Portal.

We used TCGAbiolinks^63^ using TCGAquery, TCGAdownload to obtain data from 140 GBM samples that had been profiled using Illumina platform HumanMethylation450, which interrogates 485,421 CpG sites (data level = 3, platform type = “HumanMethylation450”). We removed from the analysis data point with a corresponding *p*-value greater than 0.01, which were deemed not to carry statistically significant difference from background and defined as “NA” in TCGA level 3 data.

### HLA-I typing

The four-digit resolution HLA-I type of 812 patients (including LGG and GBM) with exome sequence bam files available in the TCGA cohort was determined using POLYSOLVER (POLYmorphic loci reSOLVER)^16^. For 647 out of 812 patients (including LGG and GBM) having RNAseq bam files available, OptiType^17^, PHLAT^18^, and seq2HLA^19^ were further applied to infer their four-digit resolution HLA-I types. The four-digit HLA-I type was determined if the predictions were consistent in any one of the following analyses: POLYSOLVER and OptiType; POLYSOLVER and PHLAT; POLYSOLVER and seq2HLA; OptiType and PHLAT; OptiType and seq2HLA.

### Neoantigen prediction

Missense mutations were used to generate a list of all possible 9-mer peptides. Binding affinities of mutant and corresponding wild-type 9-mer peptides, relevant to the patient’s HLA-I alleles, were predicted using netMHCpan-4.0^15^. High-affinity binders were defined as those with IC_50_ equal or less than 500 nM. Low-affinity wild-type binders were defined as having IC_50_ greater than 500 nM. More stringent criteria were used to infer neoantigens. A mutant-specific binder, relevant to the restricted HLA-I allele, was referred to as neoantigen when the mutant IC_50_ was less than 500 nM and IC_50_ of the corresponding wild-type binder, relevant to all HLA-I alleles of the patient, more than 500 nM. All the downstream analyses were based on the inferred neoantigens (the mutant peptides) and their corresponding wild-type peptides.

In the neoantigen fitness model, we calculated the neoantigen recognition potential (NRP) for each neoantigen using a recently developed method^27,29^. Briefly, each neoantigen was associated with a fitness cost designated as recognition potential, which is the likelihood that it is effectively recognized by the TCR repertoire. Given a neoantigen, the recognition potential was calculated as *A* × *R*. A, the amplitude, is the ratio of the relative probability that a neoantigen is bound to a class I MHC times the relative probability that the wild-type counterpart of the neoantigen is not bound

$$A = {\frac{1}{{K_d^{{\mathrm{MT}}}}}}\cdot{\frac{{K_d^{{\mathrm{WT}}}/\left[ L \right] + \epsilon(1 + K_d^{{\mathrm{WT}}}/\left[ L \right])}}{{1 + \epsilon (1 + K_d^{{\mathrm{WT}}}/\left[ L \right])}}}\; \approx \;{\frac{{K_d^{{\mathrm{WT}}}}}{{K_d^{{\mathrm{MT}}}}}}\cdot{\frac{1}{{1 + (\epsilon/\left[ L \right])K_d^{{\mathrm{WT}}}}}}$$

$$\varepsilon$$ is a pseudo-count, *K*_*d*_ is the original dissociation constant, and [*L*] the peptide concentration^29^. We set $$\varepsilon$$$$/\left[ L \right]$$ to be 0.0003^29^.

*R* is the probability that a presented neoantigen will be recognized by the TCR repertoire. We estimated *R* using a sigmoid function applied to the score of the local alignment between the peptide sequences and the set of 2552 unique epitopes in the IEDB database^29^. The sigmoid function has two parameters *a* and *k*. These parameters define the shape of the sigmoid function and are optimized as explained below. In particular, *a* representing the horizontal displacement of the binding curve, and *k* is the steepness of the curve at *a*. *A* × *R* represents the neoantigen recognition potential (NRP).

For each patient, NRP was first calculated for each neoantigen. The total neoantigen quality of a specific patient is equal to the mean value of NRPs of all neoantigens inferred for this patient.

### Parameter training

To choose the optimal model parameters *a* and *k*, we generated 4000 different *a* and *k* settings with *a* increasing from 1 to 40 at the incremental step of 1 and *k* increasing from 0.1 to 10 at the incremental step of 0.1. We selected the *a* and *k* that maximize the log-rank test scores of the survival analysis of a given patient cohort.

### Leave one out cross validation (LOOCV)

Given a patient cohort of *n* samples, each sample was sequentially removed from the set and the remaining samples (*n*–1) were used as training set on which the quality model was reoptimized. The excluded sample sequentially became the test set and was classified as high or low-quality neoantigen group. After *n* samples were sequentially classified, Kaplan–Meier analysis was applied to determine whether there was a statistically significant survival difference between high and low-quality neoantigen sub-groups.

### Random subsampling validation

Given a patient cohort, we randomly split samples into two groups in ratio of 4 vs. 1 in 100 runs. The larger group were used as training set and the smaller group as test set. The parameter settings (*a*, *k*) of the quality model were trained on the training set and tested on the test set. If samples in the test sets were separated into high and low-quality neoantigens groups with significant survival difference, the parameter setting (*a*, *k*) was successfully retained. The process was repeated 100 times to calculate the percentage of success for each parameter setting (*a*, *k*).

To calculate the significance for the success rate of each parameter setting (*a*, *k*), we performed 10,000 permutations of neoantigen qualities of patients across samples in the cohort under each parameter setting (*a*, *k*) with the random split of 4 versus 1. For each parameter setting (*a*, *k*), the success rate under each permutation was then compared with the success rate for the actual patient data. *p*-value was calculated as the percentage of permutations under which success rate was equal to or larger than the success rate of actual data.

### Neoantigen quantity model

In neoantigen quantity model, the number of inferred neoantigens was counted for each patient. Patients of a given cohort were stratified according to the mean value of the number of neoantigens into high and low neoantigen quantity groups.

### Gene signatures of immune cells

Gene signatures and relative sources of the distinct cell populations are reported in Supplementary Data 2.

### Stratification of patients based on immune cell NES and NRP

To evaluate the enrichment of each immune cell type in TCGA glioma samples we used Normalized Enrichment Score (NES) of the MWW Gene Set test^34^. NES is an estimate of the probability that the expression of a gene in the gene set is greater than the expression of a gene outside this set. Specifically,

$${NES} = 1 - \frac{U}{mn}$$

where *m* is the number of genes in a gene set, *n* the number of those outside the gene set,

$U = nm + \frac{{m(m + 1)}}{2} - T$, and *T* is the sum of the ranks of the genes in the gene set.

For each immune cell signature, the survival analysis was performed by dividing the patient cohort by the median value of NES score into low and high-immune cell groups. By intersecting high and low-quality neoantigen groups with low and high-immune cell groups information, patients were stratified into four groups (high-quality neoantigens and high-immune cells; high-quality neoantigens and low-immune cells; low-quality neoantigens and high-immune cells; low-quality neoantigens and low immune cells) and Kaplan–Meier analysis was performed to evaluate their relationship with survival.

### ee-MWW based GO enrichment analysis

To extract enriched GO categories in high-quality neoantigens and high CD8^+^ T cells compared with low-quality neoantigens and low CD8^+^ T cells, we used our recently developed ee-MWW method^34^ for comparing unbalanced datasets. The group with higher number of samples (majority class) is subsampled in order to have the same size of the subgroup with less samples (minority class), this process is repeated several times (*K* = 10,000) and the MWW test statistics is averaged across the samplings. In particular, the MWW gene-wise was applied to the two class of samples. For each sample subset *k* and the gene j, the *U*_jk_ value of the test statistic was retained. The value associate with each gene is the mean *U*_jk_ values across the *K* random subsets, $\overline {U_{\mathrm{j}}} = \frac{1}{K}{\sum}_{k = 1}^{K} U_{{\mathrm{jk}}}$. The collection of $\overline {U_j}$ values is a set of values that are the input of enrichment method NES^34^.

### Identification of differentially expressed genes

To identify genes differentially expressed between tumors in the group with high-quality neoantigens and high CD8^+^ T cells and those in the group with low-quality neoantigens and low CD8^+^ T cells from RNAseq data, we performed TCGAanalyze_DEA implementing the EdgeR protocol^66^. Multiple testing using the Benjamini–Hochberg procedure was applied to generate FDR. Genes with fold change >1.5 and FDR < 0.05 were considered as differentially expressed genes. To identify genes differentially expressed between tumors in the group with high-quality neoantigens and high CD8^+^ T cells and those in the group with low-quality neoantigens and low CD8^+^ T cells from Agilent microarray data, we used the Wilcoxon test followed by multiple testing using the Benjamini–Hochberg method for FDR estimation. Genes with fold change >1.5 and FDR < 0.05 were considered as differentially expressed genes.

### Analysis of mutations and DNA copy number changes

To define the mutations and DNA copy number changes that differentiate tumors in the group with high-quality neoantigens and high CD8^+^ T cells from tumors in the group with low-quality neoantigens and low CD8^+^ T cells, we restricted the analysis to genes with no more than one alteration in the low-quality neoantigens and low CD8 ^+^T cells group. One-sided proportion test (“greater”) was adopted to identify genes with different frequencies of mutation or copy number changes between the two groups. Genes were first ordered based on the *p*-value of the proportion test. We then selected the minimum number of genes on the basis of increasing *p*-value, so that all samples were covered by at least one alteration. The same process was used to select the genes with mutation or copy number changes specifically occurring in low-quality neoantigens and low CD8^+^ T cells group. The gene list derived from patients with both WES and RNAseq was highly overlapping with the gene list derived from patients with both WES and Agilent.

### DNA methylation analysis

Wilcoxon test followed by multiple testing using the Benjamini–Hochberg method for FDR estimation were used to identify DNA probes differentially methylated between the high-quality neoantigens and high CD8^+^ T cell group of glioma and the low-quality neoantigens and low CD8^+^ T cell group of tumors. The probes with FDR < 0.05 and absolute difference in mean methylation beta-value > 0.2 were defined as differentially methylated probes. The annotations of each Illumina platform HumanMethylation450 probe were downloaded from TCGA Data Portal website.

### Integrative expression and DNA methylation analysis

We analyzed differences in DNA methylation level between IDH wild-type GBMs groups with high CD8^+^ T cell and low CD8^+^ T cell. After removing probes not associated with promoters, the final methylation data matrix was composed of 42 IDH wild-type GBMs (17 high CD8^+^ T cell, 25 low CD8^+^ T cell samples and 85,421 probes) in the RNAseq cohort or 97 IDH wild-type GBMs with Agilent microarray data available (42 high CD8^+^ T cell, 55 low CD8^+^ T cell and 78,747 probes), respectively. Differentially DNA methylation analysis was then performed between samples with high CD8^+^ T cells versus samples with low CD8^+^ T cells using the two-sided MWW test. Differential expression analysis was performed between high versus low CD8^+^ T cell samples using edgeR and two-sided MWW test for samples with RNAseq and Agilent microarray data, respectively. Starburst plot for comparison of DNA methylation and gene expression data was constructed using the absolute value of log10(*p*-value) for differential DNA methylation (*x* axis) and gene expression *(y* axis) multiplied by the sign of the difference in methylation (*x* axis) or gene expression (*y* axis). A *p*-value less than 0.05 was considered as significant. Gene Ontology (GO) enrichment was then computed using two-sided Fisher’s exact test (FET) for a list of significant genes (hypo-methylated and upregulated genes, hyper-methylated and down-regulated genes in GBM having high CD8^+^ T cells compared with those with low CD8^+^ T cells). The significant GO terms from FET (*p*-value < 0.05; *q*-value < 0.25) were further analyzed using the Enrichment Map^67^ application of Cytoscape^68^. In the network, nodes represent the terms and edges represent known term interactions and are defined by the number of shared genes between the pair of terms. Size of the nodes is proportional to the number of genes in the category. The overlap between gene sets is computed according to the overlap coefficient (OC), defined as:

$${OC} = \frac{{\left| {{\mathbf{A}}\,{ \cap } \,{\mathbf{B}}} \right|}}{{{min}\left( {\left| {\mathbf{A}} \right|,\left| {\mathbf{B}} \right|} \right)}}$$

where **A** and **B** are two gene sets, and $\left| {\mathbf{X}} \right|$ equals to the number of elements within set **X**. We set a cutoff of ${\mathrm{OC}}\, > \, 0.5$ to select the overlapping gene sets.

### In vitro peptide-HLA I binding assay

Peptide-HLA class I in vitro binding affinities were determined as described previously^21,69,70^. Purified recombinant HLA class I heavy chains were diluted into a refolding buffer (tris-maleate buffer, pH 6.6) containing ß2m and serial 10-fold dilutions (0.018 nM to 180 µM) of the test peptide, and incubated for 48 h at 18 °C to allow for equilibrium to be reached in phosphate-buffered saline (PBS). The HLA concentration was 1.25 nM, and ß2m concentration was 10 nM. Complex formation was detected using a proximity-based luminescent oxygen channeling immunoassay. Donor beads were obtained pre-conjugated with streptavidin from Perkin Elmer; acceptor beads were conjugated in house with W6/22, a pan-specific anti-HLA class I mouse monoclonal antibody (Sigma-Aldrich) using standard procedures as described by the manufacturer. Binding affinity (Kd) was determined as described previously^21,69,70^ using the GraphPad Prism software 7.0. Data are means of counts per second. Amino acid abbreviations: A Ala; C Cys; D Asp; E Glu; F Phe; G Gly; H His; I IIe; K Lys; L Leu; M Met; N Asn; P Pro; Q Gln; R Arg; S Ser; T Thr; V Val; W Trp; Y Tyr.

### Quality model validation using an independent GBM dataset

We used whole exome sequencing data, processed mutations and updated survival information of 46 primary GBMs^30^. HLA-I subtype for each patient was obtained by mapping raw data to human reference genome hg19 using BWA aligner and applying POLYSOLVER. Neoantigen quality model with *a* = 21, *k* = 1.6 was then applied to calculate NRP for each neoantigen.

### Statistics

Comparisons between two groups were performed using an unpaired two-tailed Mann–Whitney *U*-test. Survival curves were compared using a log-rank test (Mantel-Cox). Categorical variables were compared using one-sided or two-sided Fisher exact test as indicated in figure legends. Multivariate survival analysis was performed using Cox regression model.

### Reporting Summary

Further information on experimental design is available in the Nature Research Reporting Summary linked to this article.

## Supplementary information

Reporting Summary

Supplementary Data 1

Supplementary Data 2

Supplementary Data 3

Supplementary Data 4

Supplementary Data 5

Supplementary Data 6

Supplementary Information

Description of additional supplementary items

## Data Availability

All data supporting the findings of this study are available within the published article and its supplementary information files. Figure 1 a–c have associated source data (Supplementary Table 6). All materials and other data supporting this study are available from the authors upon reasonable request.

## Code availability

We used the original codes of the authors for the computation of neoantigen quality^27^. A collection of the R procedures to perform ee-MWW and MWW-GST is available at http://github.com/miccec/yaGST^34^.
