# Supplementary material for: The combination of neoantigen quality and T lymphocyte infiltrates identifies glioblastomas with the longest survival
Source: Commun Biol. 2019 Apr 23;2:135. doi: 10.1038/s42003-019-0369-7 (PMC6478916; doi:10.1038/s42003-019-0369-7)
Supplement: Supplementary file 8 — Supplementary Information [file 42003_2019_369_MOESM8_ESM.pdf]

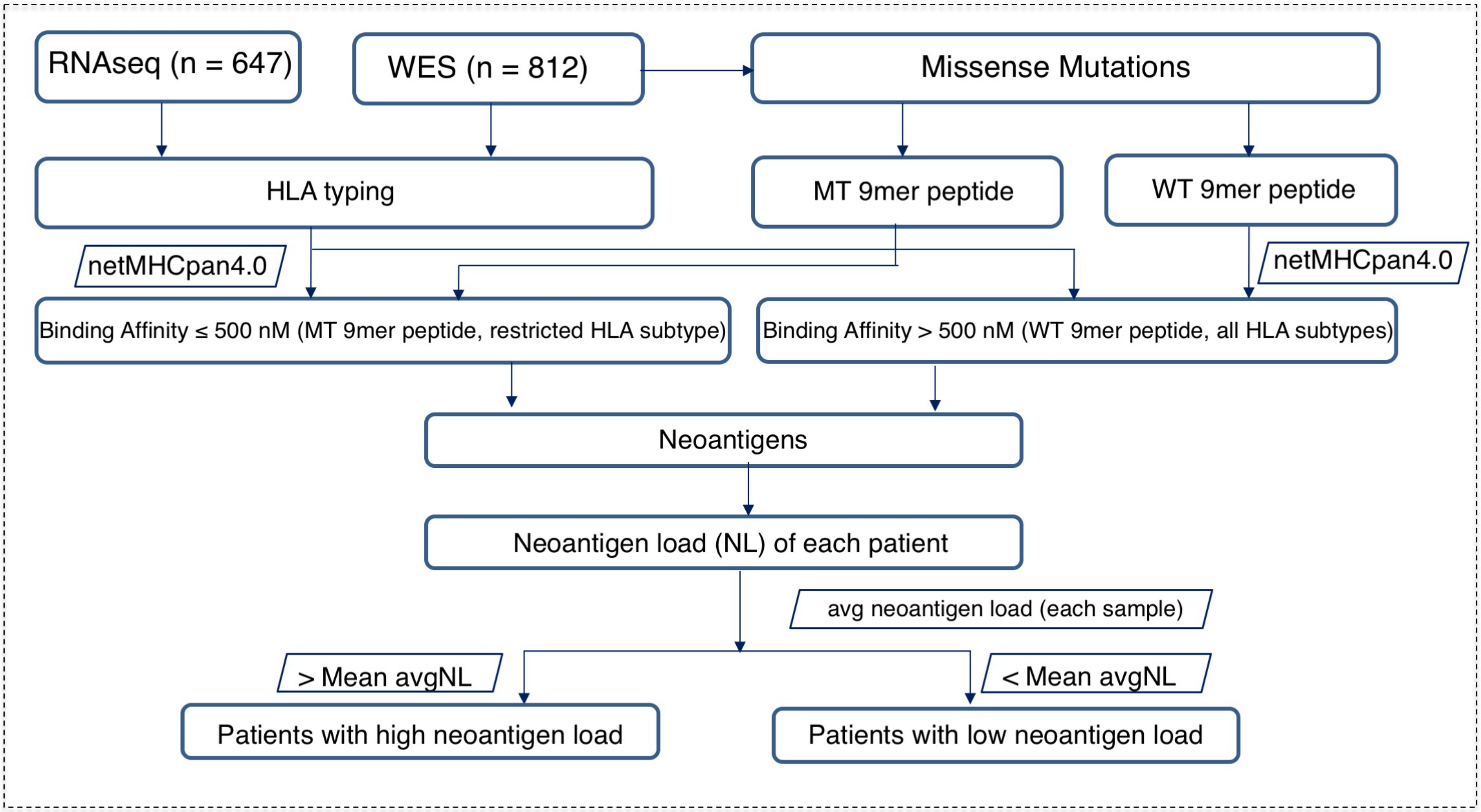

Supplementary Figure 1 Neoantigen quantity model workflow. Non-synonymous mutations were identified and used to generate a list of all possible 9-mer peptides. Binding affinities of mutant and corresponding wild type 9-mer peptides to patient HLA-I alleles were predicted using netMHCpan-4.0. High-affinity mutant binders were defined as those with an IC_{50} lower than 500 nM. Low-affinity wild type binders were defined as having IC_{50} greater than 500 nM. We considered neoantigens binders to restricted HLA-I alleles with IC_{50} of the mutant peptide <500 nM and IC_{50} of the corresponding wild type 9-mer to all HLA-I alleles of the patient >500 nM. The number of inferred neoantigens was scored for each patient. Patients in a given cohort were stratified according to the mean value of the neoantigen number into high-quantity and low-quantity neoantigen sub-groups.

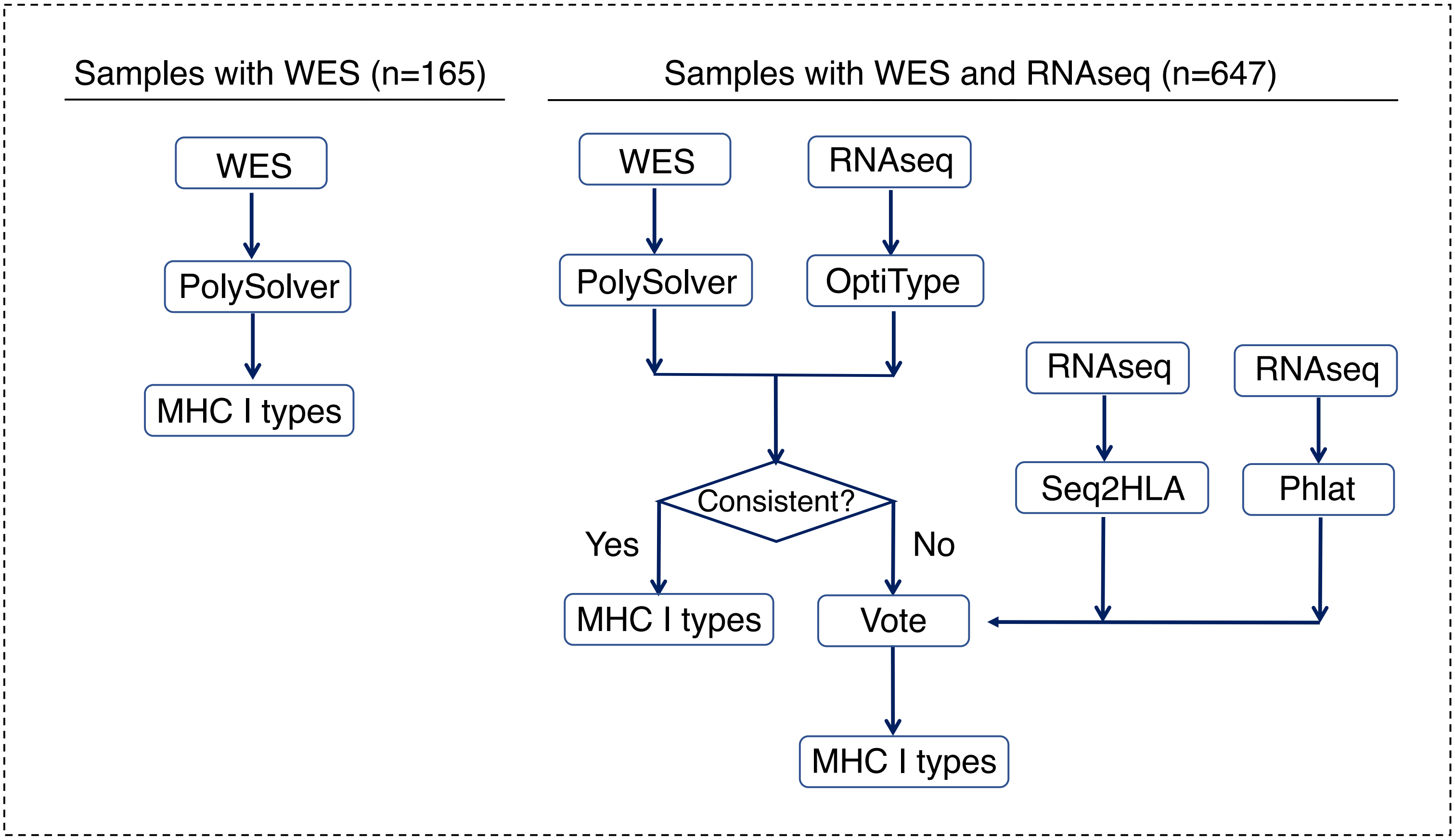

Supplementary Figure 2 HLA-I typing workflow. The four-digit resolution HLA-I type for 812 patients in the TCGA cohort (LGG plus GBM with WES data available) was determined using POLYSOLVER. The four-digit resolution HLA-I type for 647 of 812 patients having RNAseq data available was also determined using OptiType. For patients with RNAseq data available PHLAT and seq2HLA were also applied. HLA-I type was determined if the predictions were consistent in any one of the following conditions: POLYSOLVER and OptiType; POLYSOLVER and PHLAT; POLYSOLVER and seq2HLA; OptiType and PHLAT; OptiType and seq2HLA.

a

Heterozygous cases

MHC Class I	POLYSOLVER plus OptiType	OptiType plus PHLAT plus seq2HLA	OptiType plus PHLAT	OptiType plus seq2HLA	POLYSOLVER plus PHLAT	NA
HLA-A Allele 1	554/556(99.6%)	1/556 (0.2%)	1/556 (0.2%)	0	0	0
HLA-A Allele 2	481/556(86.5%)	38/556 (6.8%)	23/556 (4.1%)	7/556(1.3%)	0	7/556 (1.3%)
HLA-B Allele 1	604/607 (99.5%)	2/607 (0.3%)	1/607 (0.2%)	0	0	0
HLA-B Allele 2	543/607 (89.5%)	33/607 (5.4%)	24/607 (4%)	6/607 (1%)	0	1/607 (0.1%)
HLA-C Allele 1	577/578 (99.8%)	0	1/578 (0.2%)	0	0	0
HLA-C Allele 2	566/578 (98%)	2/578 (0.3%)	7/578 (1.2%)	0	0	3/578 (0.5%)

b

Homozygous cases

MHC Class I	POLYSOLVER plus OptiType/ POLYSOLVER plus OptiType	OptiType plus PHLATplus seq2HLA/ OptiType plus PHLAT plus seq2HLA	POLYSOLVER plus OptiType/ POLYSOLVER plus PHLAT
HLA-A	89/91 (97.8%)	1/91 (1.1%)	1/91 (1.1)

MHC Class I	POLYSOLVER plus OptiType/ POLYSOLVER plus OptiType	POLYSOLVER plus OptiType/ POLYSOLVER plus PHLAT
HLA-B	39/40 (97.5%)	1/40(2.5%)

MHC Class I	POLYSOLVER plus OptiType/ POLYSOLVER plus OptiType	POLYSOLVER plus OptiType/ POLYSOLVER plus PHLAT plus OptiType
HLA-C	68/69 (98.6%)	1/69 (1.4%)

c

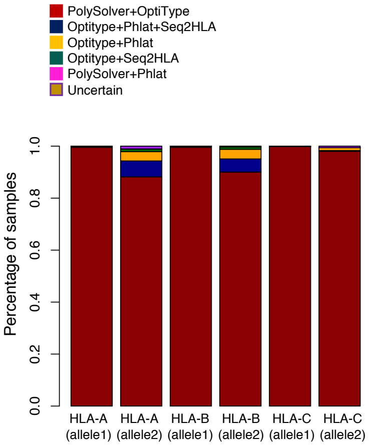

Supplementary Figure 3 HLA-I typing using the workflow in Supplementary Fig. 2. **a**, Consistency of prediction of heterozygous HLA-I alleles by POLYSOLVER, OptiType, PHLAT, and seq2HLA. **b**, Consistency of homozygous HLA-I alleles by POLYSOLVER, OptiType, PHLAT and seq2HLA. **c**, Bar graph shows the consistency of all HLA-I alleles.

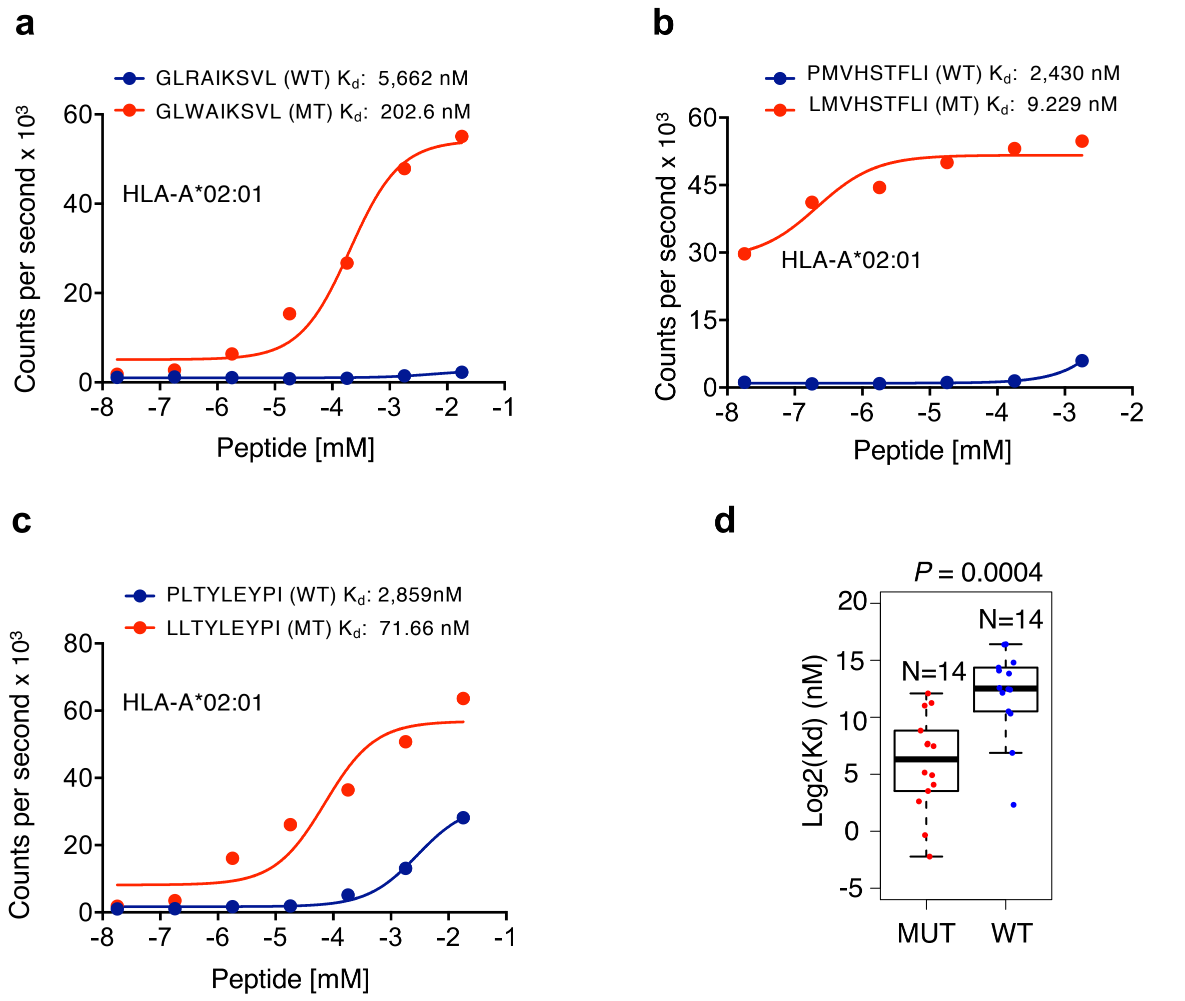

Supplementary Figure 4 *In vitro* binding affinity kinetics of neoantigens and corresponding wild type peptides for their restricted HLA class I allele. Data are shown as counts per second with increasing peptide concentration ($\log_{10}$ mM). **a**, **c**, n = 4 technical replicates from two independent experiments for wild type peptide; n = 3 technical replicates from two independent experiments for mutant peptide; **b**, n = 4 technical replicates from two independent experiments for wild type and mutant peptides. **d**, Boxplot shows *in vitro* binding affinity kinetics of 14 neoantigens and 14 corresponding wild type peptides for their restricted HLA class I allele. Boxplots show median with interquartile range; Smallest and largest observations are indicated by whiskers. *p*-value was calculated using a two-tailed Mann-Whitney U test.

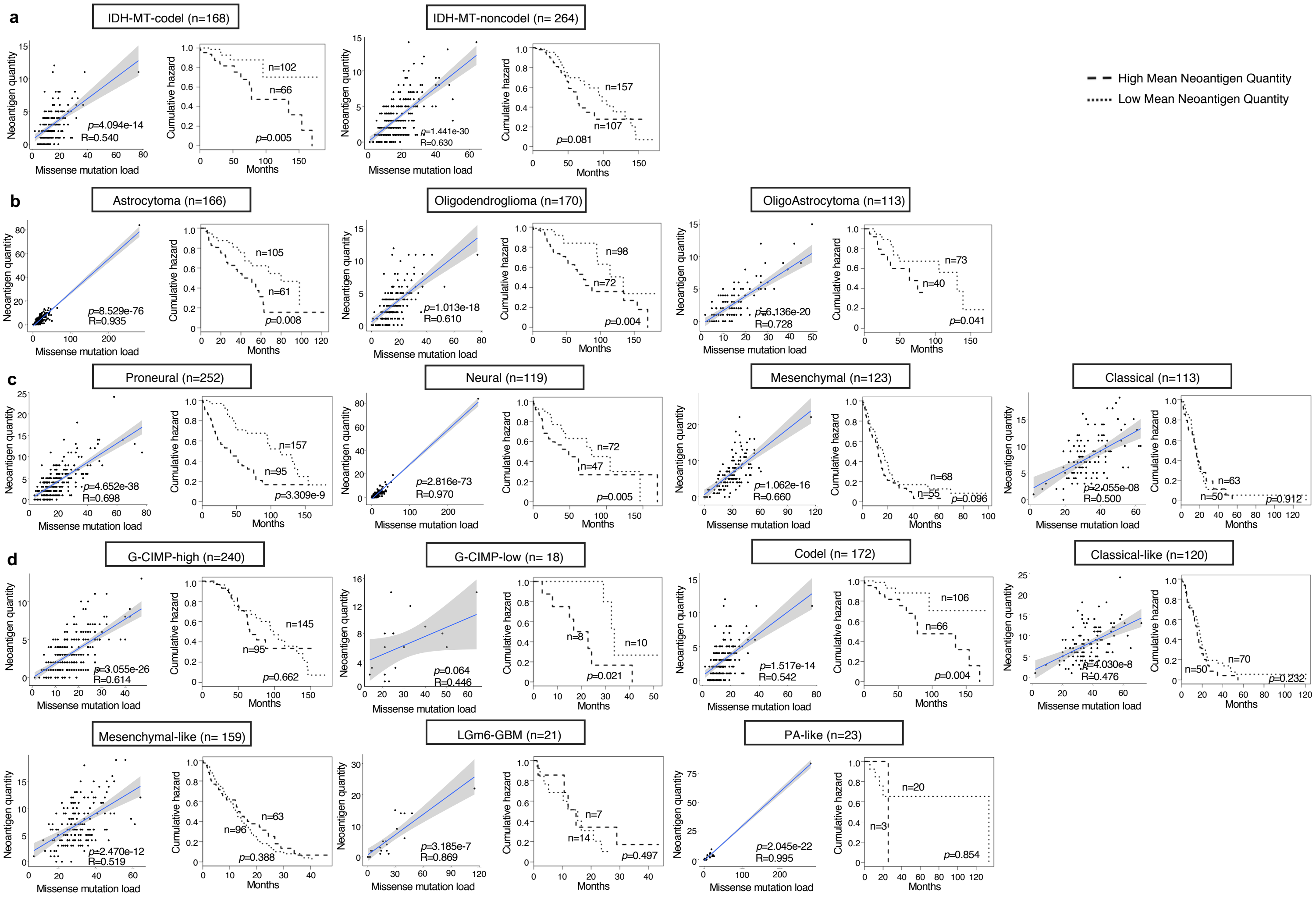

Supplementary Figure 5 Correlation between neoantigen quantity and missense mutation load and survival of glioma patients stratified according to neoantigen quantity (dashed line, high-quantity neoantigens; dotted line, low-quantity neoantigens). **a**, Glioma IDH mutant sub-groups; **b**, Glioma histology sub-groups; **c**, Glioma transcriptomic sub-groups; **d**, Glioma DNA methylation sub-groups. R, Correlation coefficient and *p*-value were calculated by Pearson correlation; n, number of patients; *p*-value for survival analysis was determined using the log-rank test.

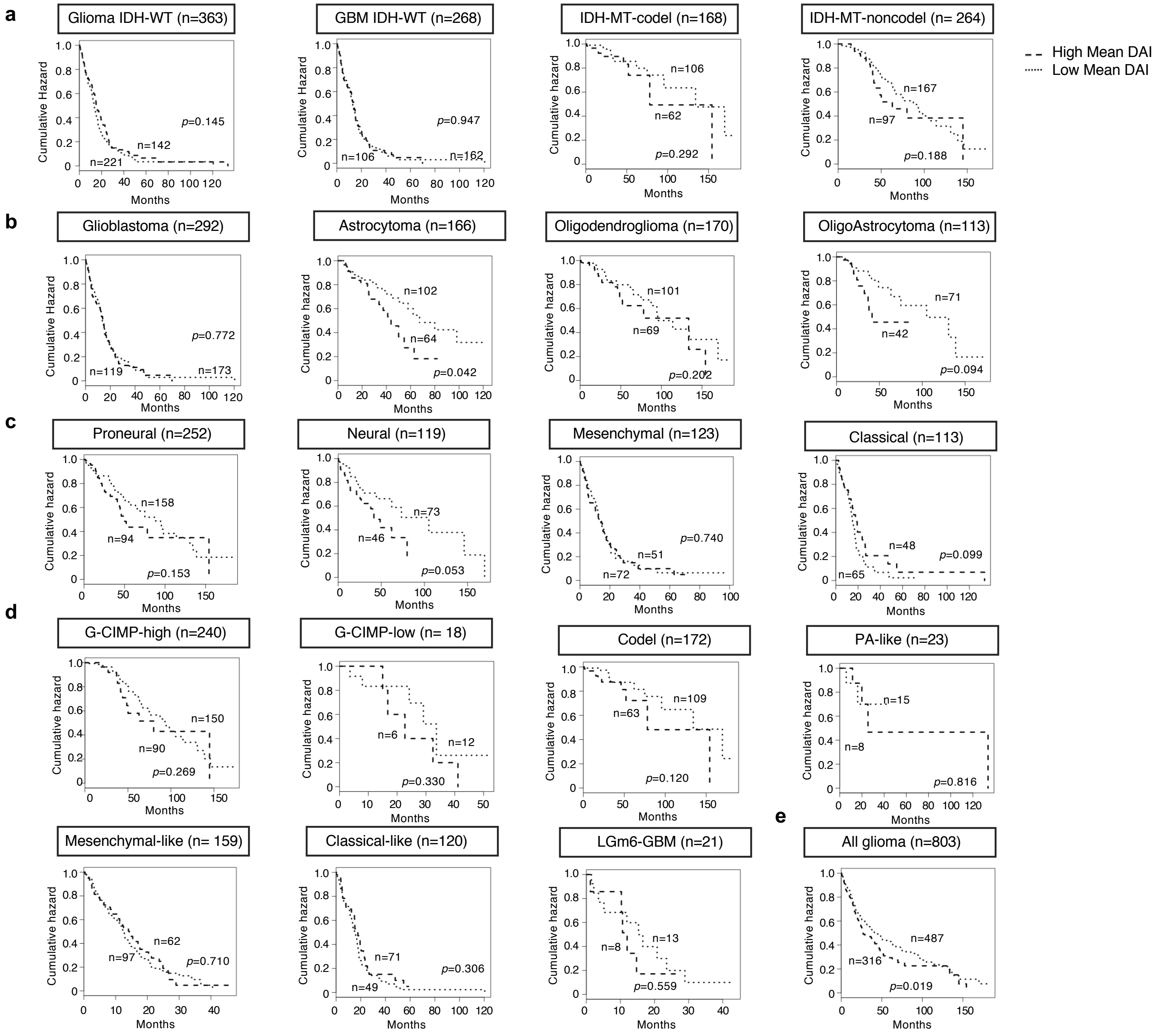

Supplementary Figure 6 Survival of glioma patients stratified according to differential agretopicity index (DAI). **a**, Glioma IDH status sub-groups; **b**, Glioma histology sub-groups; **c**, Glioma transcriptomic sub-groups; **d**, Glioma DNA methylation sub-groups. **e**, All glioma. Dashed line, high mean DAI; Dotted line, low mean DAI; n, number of patients; *p*-value was determined using the log-rank test.

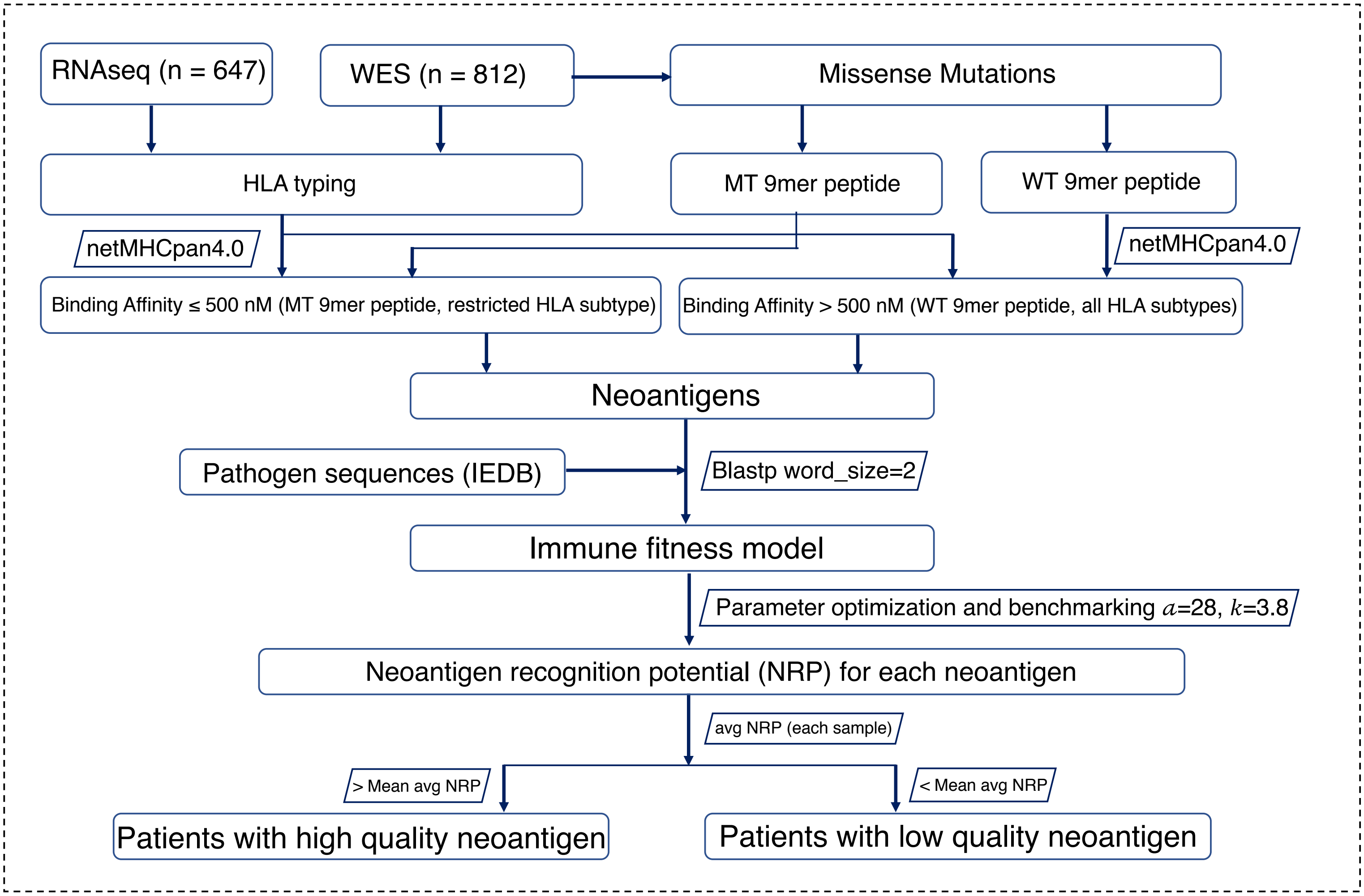

Supplementary Figure 7 Neoantigen quality model workflow. Non-synonymous mutations were identified and used to generate a list of all possible 9-mer peptides. Binding affinities of mutant and corresponding wild type 9-mer peptides to patient HLA-I alleles were predicted using netMHCpan-4.0. High-affinity mutant binders were defined as those with an IC_{50} lower than 500 nM. Low-affinity wild type binders were defined as having IC_{50} greater than 500 nM. We considered neoantigens binders to restricted HLA-I alleles with IC_{50} of the mutant peptide <500 nM and IC_{50} of the corresponding wild type 9-mer to all HLA-I alleles of the patient >500 nM. Neoantigens were used to derive alignment scores to human infectious disease-derived, class I-restricted peptide sequences showing positive immune assays in the Immune Epitope Database (IEDB). The probability of TCR recognition was then inferred using a nonlinear logistic dependence on alignment score and neoantigen recognitional potential (NRP) was calculated for each neoantigen. Neoantigen quality was computed by averaging the NRPs of neoantigens for each tumor. Patients were stratified according to the mean value of the neoantigen quality into high and low quality neoantigen sub-groups.

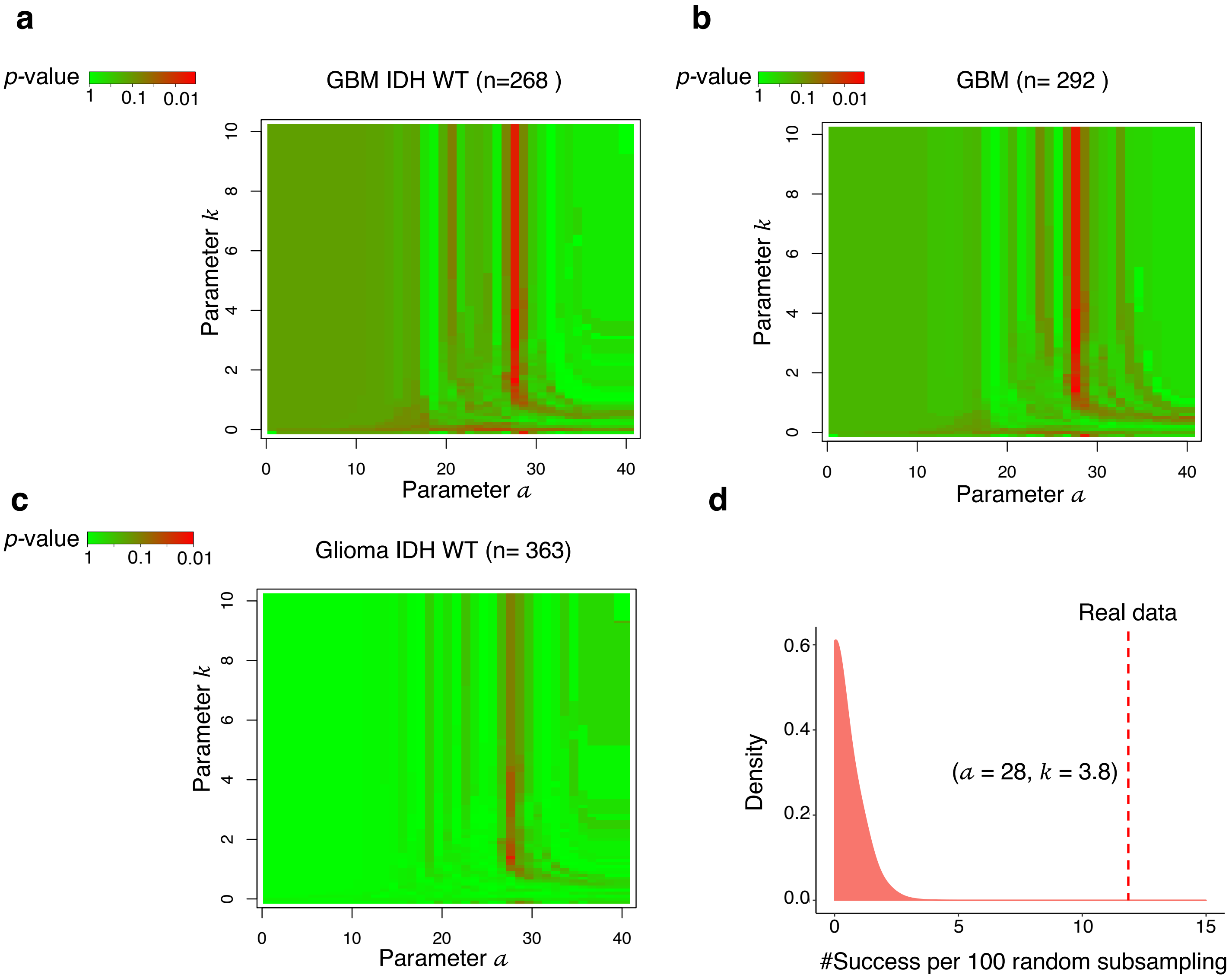

Supplementary Figure 8 Parameter optimization and benchmarking of the neoantigen quality model. The probability that a presented neoantigen is recognized by the TCR repertoire and neoantigen quality (the average NRP of neoantigens) was calculated for each patient. **a-c**, Two-dimensional histograms show the distributions of log-rank test *p*-values under different parameters of the neoantigen quality model obtained for IDH wild type GBM, GBM and IDH wild type glioma (a, horizontal displacement of the binding curve; k, steepness of the curve at a). Patients were separated into two groups on the basis of the mean value of neoantigen quality and survival between high- and low-quality neoantigen sub-groups was compared by the log-rank test. **d**, Null distribution of the number of successful separation of patients into two prognostic sub-groups per 100 random subsampling under optimal parameters (a= 28 and k = 3.8) of neoantigen quality model. n, number of patients. *p*-value was determined using the log-rank test and is shown as color bar.

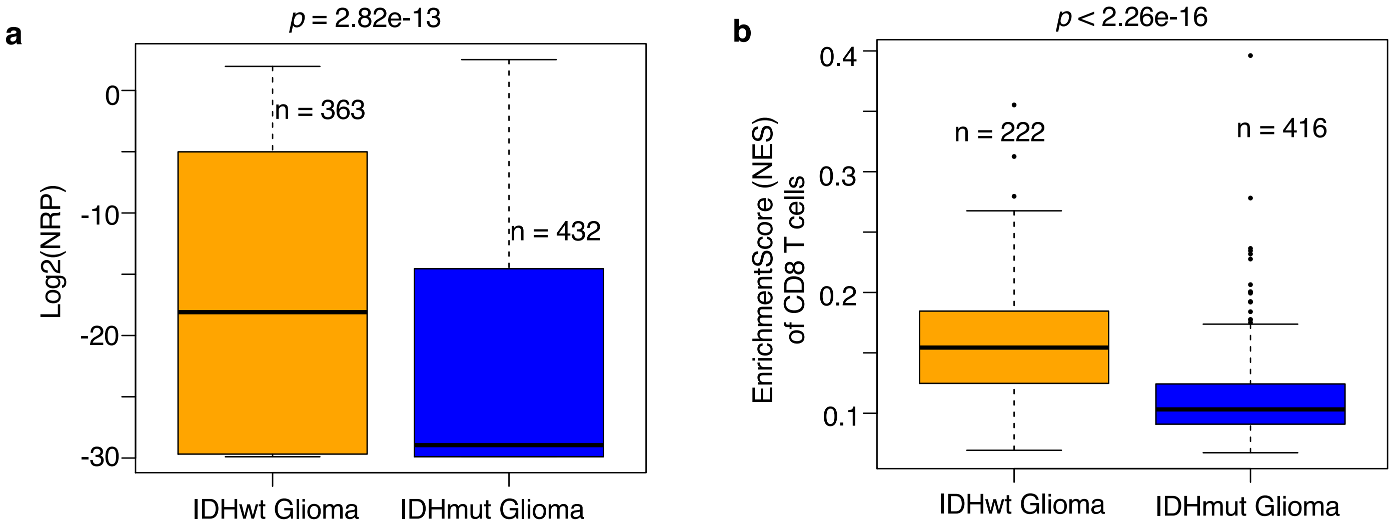

Supplementary Figure 9 Comparison of NRP and CD8$^+$T cell enrichment scores between IDH wild type and IDH mutant gliomas. **a**, Log2 transformed NRP was compared between IDH wild type and IDH mutant gliomas. **b**, CD8$^+$ T cell enrichment score (NES) was compared between IDH wild type and IDH mutant gliomas. n, number of patients. NRP, neoantigen recognition potential score. n, number of patients. Boxplots show median with interquartile range; Smallest and largest observations are indicated by whiskers. *p*-value was calculated using a two-tailed Mann-Whitney U test.

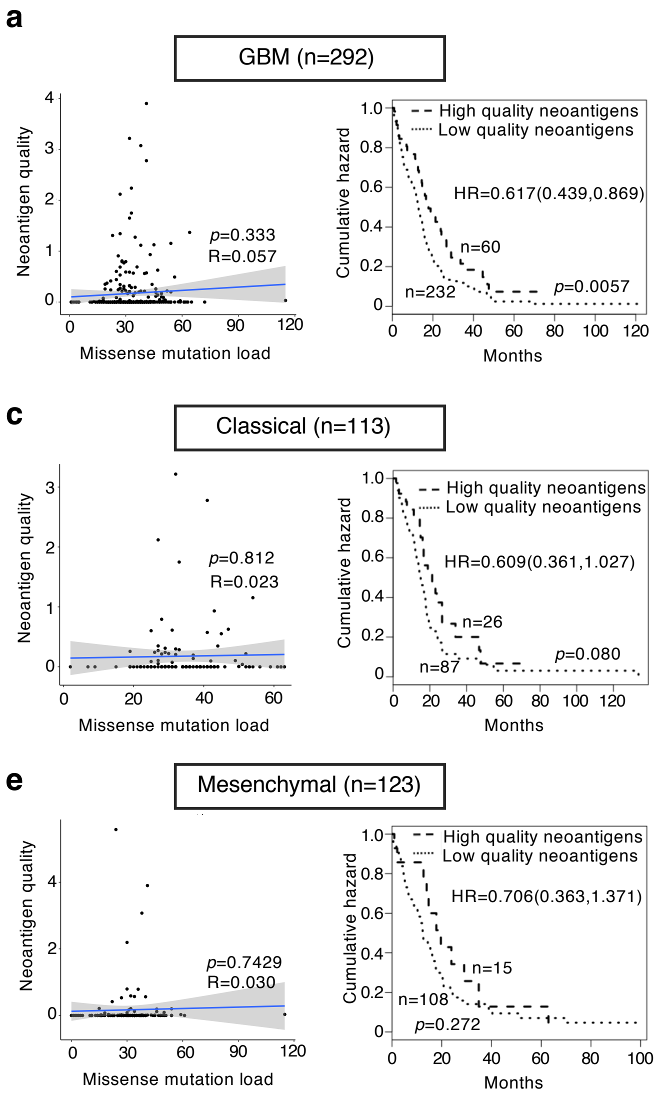

Supplementary Figure 10 Correlation between neoantigen quality and missense mutation load (left panels) and survival of glioma patients stratified according to neoantigen quality (right panels; dashed line, high quality neoantigens; dotted line, low quality neoantigens). **a**, GBM; **b**, Glioma IDH wild type; **c**, Classical; **d**, Classic-like; **e**, Mesenchymal. n, number of patients. *p*-values was determined using the log-rank test.

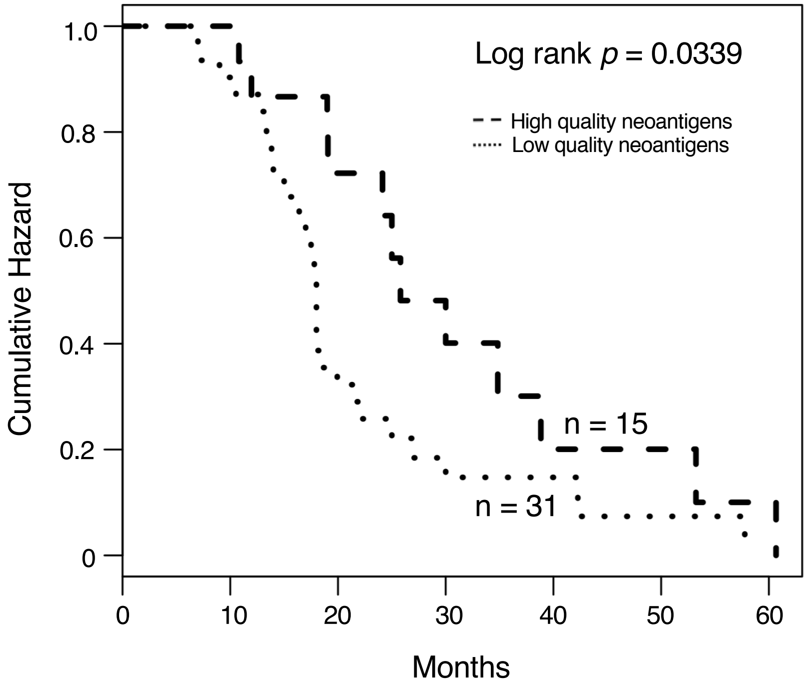

Supplementary Figure 11 Validation of the high-quality neoantigens model as a prognostic predictor in primary GBM using an independent data set of 46 patients; log rank test p=0.0339; n, number of patients.

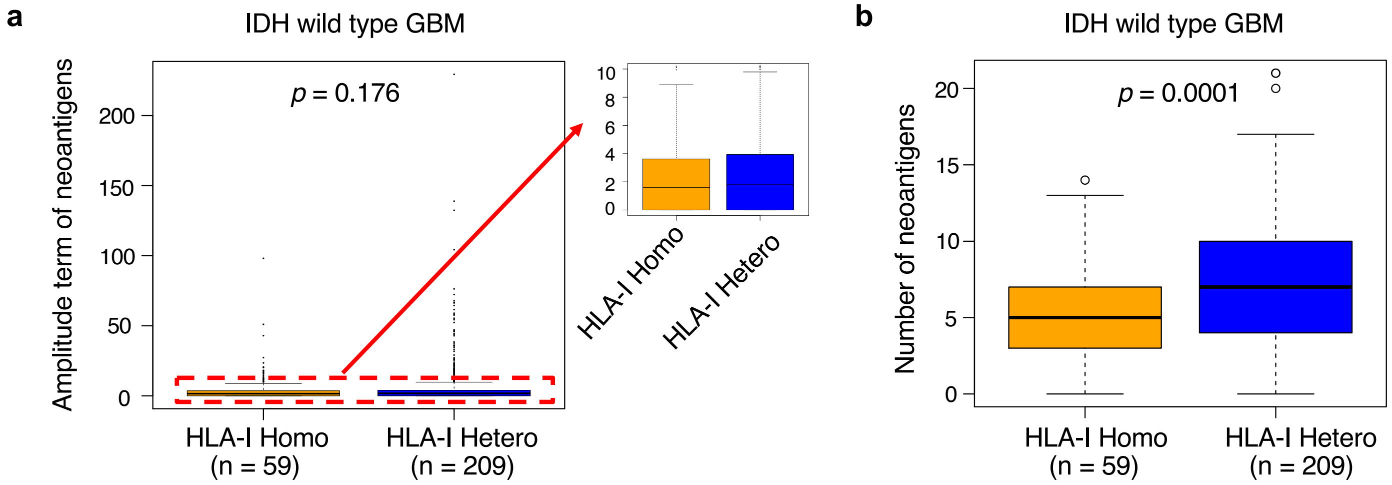

Supplementary Figure 12 Comparison of the amplitude term (**a,** left panel) and the number of neoantigens (**b**) between HLA-I homozygosity and HLA-I heterozygosity in IDH wild type GBMs. Right panel in a is the expanded boxplot with median and interquartile range. n, number of patients. *p*-value was determined using Mann Whitney U test. Boxplots show median with interquartile range; Smallest and largest observations are indicated by whiskers. *p*-value was calculated using a two-tailed Mann-Whitney U test.

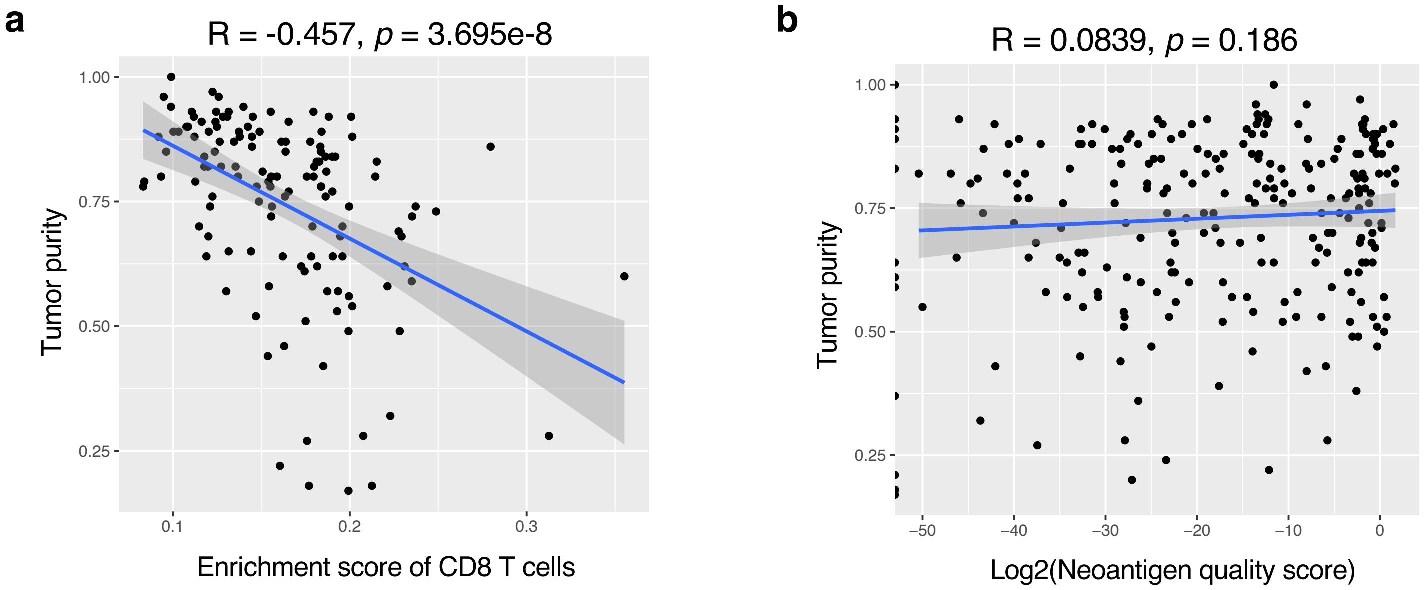

Supplementary Figure 13 Analysis of tumor purity and immune characteristics of IDH wild type GBM. **a**, Pearson correlation between tumor purity evaluated using ABSOLUTE and $CD8^+$ T cell enrichment. **b**, The Pearson correlation between tumor purity and neoantigen quality score. *p*-value was calculated using Pearson correlation; n, number of patients

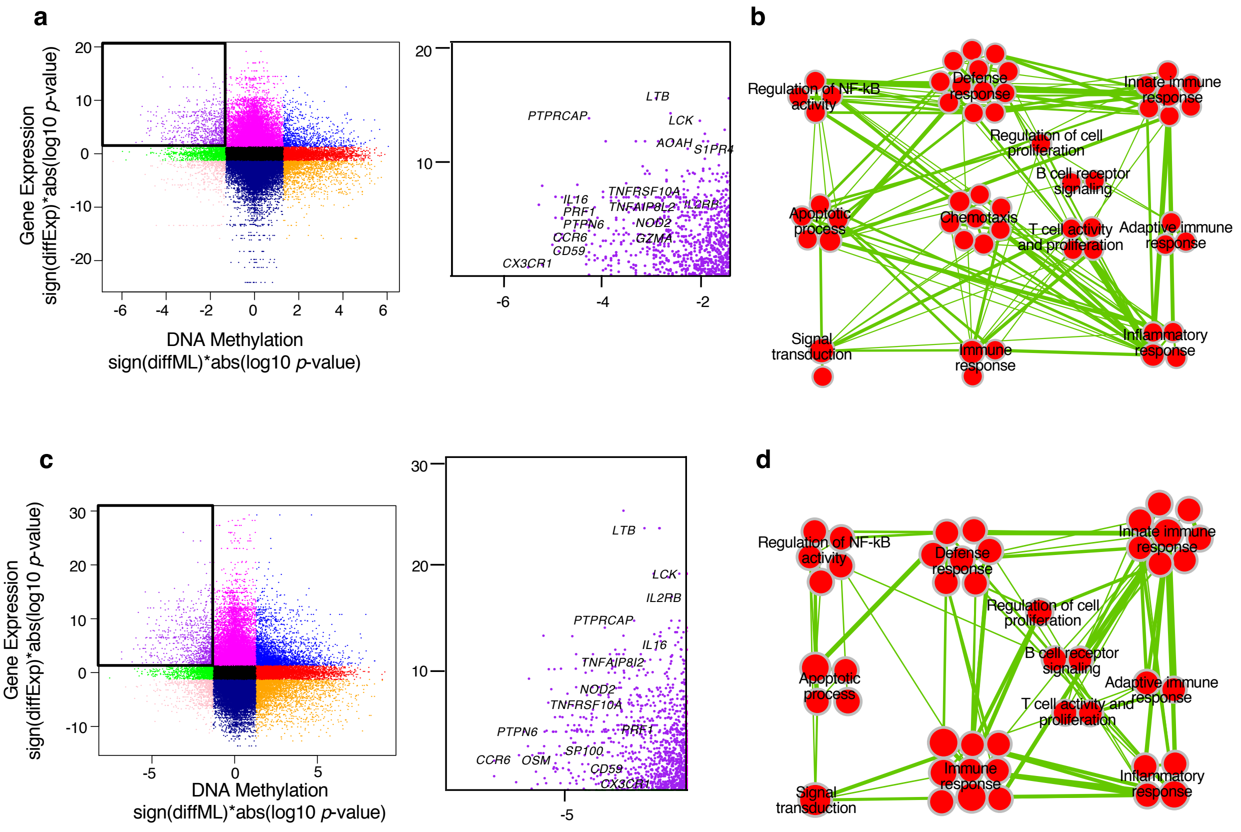

Supplementary Figure 14 Integrative analysis of gene expression and DNA methylation in high CD8+ T cells IDH wild type GBM. **a. c.** Starburst plot of supervised differential gene expression and DNA methylation between samples with high CD8+ T cells versus samples with low CD8+ T cells (RNAseq for 17,301 unique genes or Agilent expression array data for 17,814 unique genes in **a** and **c**, respectively). *x* axis, absolute value of log10(p-value) of differential methylation multiplied by the sign of the differential methylation; *y* axis, absolute value of log10(p-value) of differential gene expression multiplied by the sign of differential expression. The top left area of each plot corresponding to the significantly hypo-methylated and up-regulated genes in samples with high CD8+ T cells compared with samples with low CD8+ T cell is magnified in the right panels and genes involved in immune response and T cell functions are indicated. **b. d.** Enrichment map network of statistically significant GO categories (two-sided Fisher's exact test $p < 0.05$) for the lists of hypo-methylated and up-regulated genes in high CD8+ T cell compared with low CD8+ T cell samples (RNAseq or Agilent expression array data in **b** and **d**, respectively). Nodes represent GO terms and lines their connectivity. Node size is proportional to number of genes in the GO category and line thickness indicates the fraction of genes shared between groups.

Supplementary Table 1. Multivariate Cox regression analysis including high neoantigen quality, mutation load, age and gender for IDH wild type GBM (n=268).

	HR	95%CI	*P*
Neoantigen quality (high)	0.61152	[0.4301,0.8695]	0.00617
Age	1.032035	[1.0184,1.0458]	3.29E-06
Gender	1.242203	[0.9292,1.6607]	0.14319
Mutation load	0.989501	[0.9772,1.0020]	0.09849

Supplementarty Table 2. Distribution of HLA-I homozygosity and heterozygosity for high and low quality neoantigens in IDH wild type GBM.

Patients with WES	High quality neoantigen IDH-WT GBM (n=56)	Low quality neoantigen IDH-WT GBM (n=212)	*p*-value
Homogyzosity	6 (10.7%)	53(25.0%)	0.0136
Heterozygosity	50(89.3%)	159(75.0%)	n.s.

p-value was determined by one-sided Fisher exact test.

Supplementary Table 3. Prognostic significance of tumor infiltrating cell types and high-quality neoantigens in IDH wild type GBM.

Cell Type	Log rank P value (RNAseq)	Log rank P value (Agilent)	Neoantigens + Cells	Log rank P value (RNAseq)	Log rank P value (Agilent)
CD8 T cells	0.1343	0.1357	Neoantigens + CD8 T cells	0.0016	0.0048
Blood-derived Macrophages	0.3876	0.2035	Neoantigens + Blood-derived Macrophages	0.1325	0.2503
Microglia	0.5929	0.7134	Neoantigens + Microglia	0.1848	0.0241
Oligodendrocyte	0.0107	0.0912	Neoantigens + Oligodendrocyte	0.9694	0.4017
Endothelial	0.7235	0.0078	Neoantigens + Endothelial	0.0379	0.00036
Pericytes	0.991	0.1415	Neoantigens + Pericytes	0.0206	0.0043
Interferon Gamma	0.1304	0.2547	Neoantigens + Interferon Gamma	0.0008	0.0108
IFNG	0.1881	0.4953	Neoantigens + IFNG	0.0012	0.0135
Exhausted CD8 T cell (Up-regulated)	0.6833	0.8724	Neoantigens + Exhausted CD8 T cell (Up-regulated)	0.1012	0.1146
Exhausted CD8 T cell (Down-regulated)	0.74	0.1713	Neoantigens + Exhausted CD8 T cell (Down-regulated)	0.0193	0.0025
CD8 TRM	0.4508	0.9642	Neoantigens + CD8 TRM	0.2101	0.1098
CD8 TRM mitotic	0.397	0.1626	Neoantigens + CD8 TRM mitotic	0.0324	0.0068
CD8 TEM	0.9067	0.2929	Neoantigens + CD8 TEM	0.0749	0.0089
CD8 GD	0.5552	0.3712	Neoantigens + CD8 GD	0.0398	0.0067
CD4 TRM	0.6268	0.2055	Neoantigens + CD4 TRM	0.1124	0.4092
CD4 RGCC	0.8792	0.7594	Neoantigens + CD4 RGCC	0.021	0.0287
CD4 IL7R	0.5686	0.5627	Neoantigens + CD4 IL7R	0.031	0.0447
CD4 FOXP3	0.4935	0.6902	Neoantigens + CD4 FOXP3	0.0688	0.0509
CD4 CXCL13	0.6931	0.8091	Neoantigens + CD4 CXCL13	0.0778	0.1435
Monocytes	0.1889	0.7659	Neoantigens + Monocytes	0.2618	0.0884

Supplementary Table 4. Multivariate Cox regression analysis of high quality neoantigen and high CD8 T cells, clinical, and mutation load in IDH wild type GBM.

Supplementary Table 4a. Multivariate Cox regression analysis of neoantigen quality (high) and CD8 T cells (high), age, gender, and mutation load in IDH wild type GBM with both WES and RNAseq data available.

Multivariate Cox regression analysis of IDH wild type GBM (covered by RNAseq Data, N=67*)

	HR	95%CI	P
Neoantigen quality (high) & CD8 T cells (high)	0.51177	[0.3332, 0.7860]	0.00222
Age	1.05185	[1.0126, 1.0926]	0.00918
Gender	0.83574	[0.3571, 1.9557]	0.67912
Mutation load	0.95804	[0.9239, 0.9935]	0.02076

* IDH wild type GBM with high quality neoantigen & high CD8 T cells and IDH wild type GBM with low quality neoantigen & low CD 8 T cells

Supplementary Table 4. Multivariate Cox regression analysis of high quality neoantigen and high CD8 T cells, clinical, and mutation load in IDH wild type GBM.
Supplementary Table 4b. Multivariate Cox regression analysis of neoantigen quality (high) and CD8 T cells (high), age, gender, and mutation load in IDH wild type GBM with both WES and Agilent data available.

Multivariate Cox regression analysis of IDH wild type GBM (covered by Agilent Data, N=125*)

	HR	95%CI	P
Neoantigen quality (high) & CD8 T cells (high)	0.74058	[0.5535, 0.9909]	0.0432
Age	1.06983	[1.0355, 1.1052]	4.86E-05
Gender	1.90888	[1.0030, 3.6329]	0.0489
Mutation load	0.96563	[0.9366, 0.9955]	0.09849

* IDH wild type GBM with high quality neoantigen & high CD8 T cells and IDH wild type GBM with low quality neoantigen & low CD 8 T cells

Supplmentary Table 5. The frequency of the combined somatic alterations of PIK3CA, RB1 and MDM2.

Supplementary Table 5a. Somatic alterations of *PIK3CA*, *RB1* and *MDM2* in patients having both high quality neoantigens and high CD8 T lymphocytes or patients having both low quality neoantigens and low CD8 T lymphocytes (IDH wild type GBM cohort analyzed using WES and RNAseq). The *p*-value was determined by one-sided Fisher's exact test.

RNAseq	SNV-CNV (PIK3CA, RB1, MDM2)	No SNV-CNV (PIK3CA, RB1,MDM2)	*p*-value
Low quality neoantigens and low CD8 T cells	13 (23.6%)	42 (76.4%)	0.0572
High quality neoantigens and high CD8 T cells	0	12 (100%)	

Supplmentary Table 5. The frequency of the combined somatic alterations of PIK3CA, RB1 and MDM2.

Supplementary Table 5b. Somatic alterations of *PIK3CA*, *RB1* and *MDM2* in patients having both high quality neoantigens and high CD8 T lymphocytes or patients having both low quality neoantigens and low CD8 T lymphocytes (IDH-WT GBM cohort analyzed using WES and Agilent microarrays). The *p*-value was determined by one-sided Fisher's exact test.

Agilent microarray	SNV-CNV (PIK3CA, RB1, MDM2)	No SNV-CNV (PIK3CA, RB1,MDM2)	*p*-value
Low quality neoantigens and low CD8 T cells	28 (28.3%)	71 (71.7%)	0.0207
High quality neoantigens and high CD8 T cells	2 (7.7%)	24 (92.31%)	

Supplementary Table 6. Source Data for Figure 1

Figure 1 a

Allele	HLA-B*07:02	HLA-B*07:02	HLA-B*07:02	HLA-B*07:02	HLA-B*07:02	HLA-B*07:02
PeptideSequence	GSLCHATAM	GSLCHATAM	GSLCHATAM	GSLCHATAM	RSLCHATAM	RSLCHATAM
ExperimentBatch	1	1	2	2	1	1
PeptideInfo	WT	WT	WT	WT	Mut	Mut
-log10(mM)	Affinity	Affinity	Affinity	Affinity	Affinity	Affinity
-7.744728	0.476	0.526	0.437	1.864	2.232	4.649
-6.744728	0.682	0.951	0.291	0.956	2.78	1.911
-5.744728	0.665	1.245	0.502	0.603	11.04	15.87
-4.744728	1.284	2.669	2.514	0.775	61.173	55.133
-3.744728	4.089	6.787	16.162	4.969	90.294	85.145
-2.744728	16.238	22.627	18.358	22.925	123.882	108.512
-1.744728	31.414	47.699	48.653	66.886	138.194	140.843
-0.7447275	45.456	64.972	61.416	79.222	139.331	133.171

Figure 1 b

Allele	HLA-B*07:02	HLA-B*07:02	HLA-B*07:02	HLA-B*07:02	HLA-B*07:02	HLA-B*07:02	HLA-B*07:02	HLA-B*07:02	HLA-B*07:02	HLA-B*07:02
PeptideSequence	VAEGQTLDL	VAEGQTLDL	VAEGQTLDL	VAEGQTLDL	VPEGQTLDL	VPEGQTLDL	VPEGQTLDL	VPEGQTLDL	VPEGQTLDL	VPEGQTLDL
ExperimentBatch	1	1	2	2	1	1	2	2	2	2
PeptideInfo	WT	WT	WT	WT	Mut	Mut	Mut	Mut	Mut	Mut
-log10(mM)	Affinity	Affinity	Affinity	Affinity	Affinity	Affinity	Affinity	Affinity	Affinity	Affinity
-7.744728	0.555	0.626	0.578	0.681	1.785	0.926	1.2	0.672	1.211	1.753
-6.744728	0.983	1.087	0.621	0.573	1.988	0.585	1.952	0.479	2.218	2.371
-5.744728	1.242	2.507	0.401	2.996	21.846	16.367	16.585	16.559	22.622	14.809
-4.744728	1.393	1.297	0.517	1.332	51.778	39.566	51.71	49.233	54.426	63.84
-3.744728	1.561	1.562	0.692	1.534	91.351	96.64	99.28	103.467	105.363	104.897
-2.744728	2.411	2.437	0.951	2.258	133.071	116.131	126.564	111.613	118.572	122.317
-1.744728	7.984	9.973	4.174	6.574	132.782	132.002	148.272	139.55	133.503	149.845
-0.7447275	23.167	26.126	22.967	27.69	133.148	129.239	138.601	128.208	129.227	141.846

Figure 1 c

Allele	HLA-B*07:02	HLA-B*07:02	HLA-B*07:02	HLA-B*07:02	HLA-B*07:02	HLA-B*07:02
PeptideSequence	LLWKTWYPC	LLWKTWYPC	LLWKTWYPC	LLWKTWYPC	LPWKTWYPC	LPWKTWYPC
ExperimentBatch	1	1	2	2	1	1
PeptideInfo	WT	WT	WT	WT	Mut	Mut
-log10(mM)	Affinity	Affinity	Affinity	Affinity	Affinity	Affinity
-7.744728	0.888	0.886	0.441	0.768	2.174	1.745
-6.744728	1.009	0.947	0.447	0.456	4.324	1.529
-5.744728	0.974	1.35	0.47	0.529	20.865	15.9
-4.744728	1.189	1.237	0.612	0.672	81.095	71.419
-3.744728	1.57	1.558	0.82	0.805	110.124	109.396
-2.744728	4.237	3.851	2.195	3.436	144.338	129.504
-1.744728	15.091	14.028	14.533	12.507	131.99	136.471

Supplementary References

1 Ceccarelli, M. *et al.* Molecular Profiling Reveals Biologically Discrete Subsets and Pathways of Progression in Diffuse Glioma. *Cell* **164**, 550-563, doi:10.1016/j.cell.2015.12.028 (2016).
2 Yuan, J. *et al.* Single-cell transcriptome analysis of lineage diversity in high-grade glioma. *Genome Med* **10**, 57, doi:10.1186/s13073-018-0567-9 (2018).
3 Jamieson, N. B. & Maker, A. V. Gene-expression profiling to predict responsiveness to immunotherapy. *Cancer Gene Ther* **24**, 134-140, doi:10.1038/cgt.2016.63 (2017).
4 Ayers, M. *et al.* IFN-gamma-related mRNA profile predicts clinical response to PD-1 blockade. *J Clin Invest* **127**, 2930-2940, doi:10.1172/JCI91190 (2017).
5 Bengsch, B. *et al.* Epigenomic-Guided Mass Cytometry Profiling Reveals Disease-Specific Features of Exhausted CD8 T Cells. *Immunity* **48**, 1029-1045 e1025, doi:10.1016/j.immuni.2018.04.026 (2018).
6 Savas, P. *et al.* Single-cell profiling of breast cancer T cells reveals a tissue-resident memory subset associated with improved prognosis. *Nat Med* **24**, 986-993, doi:10.1038/s41591-018-0078-7 (2018).
7 Hendrickx, W. *et al.* Identification of genetic determinants of breast cancer immune phenotypes by integrative genome-scale analysis. *Oncoimmunology* **6**, e1253654, doi:10.1080/2162402X.2016.1253654 (2017).
