# Supplementary material for: The combination of neoantigen quality and T lymphocyte infiltrates identifies glioblastomas with the longest survival
Source: Commun Biol. 2019 Apr 23;2:135. doi: 10.1038/s42003-019-0369-7 (PMC6478916; doi:10.1038/s42003-019-0369-7)
Supplement: Supplementary file 9 — Description of additional supplementary items [file 42003_2019_369_MOESM9_ESM.docx]

**Description of additional supplementary files**

**File Name**: Supplementary Data 1

**Description:** Characteristics of TCGA pan-glioma cohort including Supplementary Data 1a and 1b. Supplementary Data 1a. Classification of glioma patients according to histology, IDH status, transcriptome subtype, and DNA methylation cluster. Supplementary Data 1b. Clinical and molecular characteristics of glioma patients and the genomic platforms analyzed.

**File Name:** Supplementary Data 2

**Description:** The list of genes used for the characterization of tumor infiltrating cell types and corresponding references.

**File Name:** Supplementary Data 3

**Description:** Supplementary Data 3. The lists of differentially methylated and differentially expressed genes in glioma analyzed by RNAseq including Supplementary Data 3a and 3b. Supplementary Data 3a. The lists of hypo-methylated and up-regulated genes in tumors with high CD8 T cells compared with tumors with low CD8 T cells. Supplementary Data 3b. The lists of hyper-methylated and down-regulated genes in tumors with high CD8 T cells compared with tumors with low CD8 T cells.

**File Name:** Supplementary Data 4

**Description:** Supplementary Data 4. The lists of differentially methylated and differentially expressed genes in tumors analyzed by Agilent microarray including Supplementary Data 4a and 4b. Supplementary Data 4a. The lists of hypo-methylated and up-regulated genes in tumors having high CD8 T cells compared with tumors with low CD8 T cells. Supplementary Data 4b. The lists of hyper-methylated and down-regulated genes in tumors with high CD8 T cells compared with tumors having low CD8 T cells.

**File Name:** Supplementary Data 5

**Description:** Supplementary Data 5. Functional annotation for the lists of genes differentially methylated and differentially expressed in tumors analyzed by RNAseq including Supplementary Data 5a and 5b. Supplementary Data 5a. Functional annotation for the lists of hypo-methylated and up-regulated genes in tumors having high CD8 T cells compared with tumors with low CD8 T cells. Supplementary Data 5b. Functional annotation for the lists of hyper-methylated and down-regulated genes in tumors having high CD8 T cells compared with tumors with low CD8 T cells.

**File Name:** Supplementary Data 6

**Description:** Supplementary Data 6. Functional annotation for the lists of differentially methylated and differentially expressed genes in tumors analyzed by Agilent microarrays including Supplementary Data 6a and 6b. Supplementary Data 6a. Functional annotation for the lists of hypo-methylated and up-regulated genes in glioma having high CD8 T cells compared with tumors with low CD8 T cells. Supplementary Data 6b. Functional annotation for the lists of hyper-methylated and down-regulated genes in tumors having high CD8 T cells compared with tumors with low CD8 T cells.
